# In Vitro Study on the Effects of *Rhododendron mucronulatum* Branch Extract, Taxifolin-3-O-Arabinopyranoside and Taxifolin on Muscle Loss and Muscle Atrophy in C2C12 Murine Skeletal Muscle Cells

**DOI:** 10.3390/ijms27020570

**Published:** 2026-01-06

**Authors:** Hyun Seo Lee, Hyeon Du Jang, Tae Hee Kim, Da Hyeon An, Ye Eun Kwon, Eun Ji Kim, Jae In Jung, Sangil Min, Hee Kyu Kim, Kwang-Hyun Park, Heesung Woo, Sun Eun Choi

**Affiliations:** 1 Department of Forest Biomaterials Engineering, Kangwon National University, Chuncheon 24341, Gangwon State, Republic of Korea; hyunseo2002@kangwon.ac.kr (H.S.L.); wkdgusen98@naver.com (H.D.J.); 202416560@kangwon.ac.kr (D.H.A.); 2 Dr.Oregonin Inc., #802 Bodeum Hall, Kangwondaehakgil 1, Chuncheon 24341, Gangwon State, Republic of Korea; kth02120@naver.com (T.H.K.); kye0519@naver.com (Y.E.K.); 3 Department of AI Biological Parts Materials Engineering, Nambu University, Gwangju Metropolitan City 62271, Republic of Korea; 4 Research Institute, SRE Service Co., Ltd., Chuncheon 24232, Gangwon State, Republic of Korea; myej44@naver.com; 5 Institute of Medical Bio-Convergence, Hallym University, Chuncheon 24252, Gangwon State, Republic of Korea; jungahoo@hallym.ac.kr; 6 Division of Transplantation and Vascular Surgery, Department of Surgery, Seoul National University Hospital, Seoul 03080, Republic of Korea; surgeonmsi@gmail.com; 7 Gangwon State Forest Science Institute, 24, Hwamokwon-gil, Chuncheon 24207, Gangwon State, Republic of Korea; dearkyu@korea.kr; 8 Department of Emergency Medical Rescue, Nambu University, Gwangju Metropolitan City 62271, Republic of Korea; khpark@nambu.ac.kr; 9 Department of Forest Engineering, Resources and Management, Oregon State University, 352 Peavy Forestry Science Center, Corvallis, OR 97331-5703, USA; heesung.woo@oregonstate.edu

**Keywords:** *Rhododendron mucronulatum*, taxifolin-3-O-arabinopyranoside, taxifolin, apoptosis, muscle atrophy, sarcopenia

## Abstract

Sarcopenia, an age-related muscle atrophy disease, is a major health concern in aging societies and is closely associated with severe chronic diseases. Its primary pathogenesis involves oxidative stress-induced apoptosis in muscle cells and an imbalance in protein metabolism. This study evaluated the potential of *Rhododendron mucronulatum* branch extract (RMB) and its major flavonoids, taxifolin-3-O-arabinopyranoside (Tax-G) and taxifolin (Tax-A), as natural therapeutic agents for sarcopenia. Phytochemical analyses were performed using TLC, HPLC, LC-MS/MS, and NMR, and Tax-G and Tax-A were isolated from RMB. In vitro models of apoptosis and muscle atrophy were established in C2C12 cells using H_2_O_2_ and dexamethasone (DEX), respectively. Cell viability, myotube diameter, and protein expression related to apoptosis and muscle differentiation were assessed. All three substances reduced H_2_O_2_-induced apoptosis by increasing Bcl-2 and inhibiting cleaved caspase-3 and PARP. They also attenuated DEX-induced muscle atrophy by suppressing Atrogin-1, MuRF1, and FoxO3α while promoting MyoD, Myogenin, Akt, and mTOR. Although Tax-A showed the highest activity, Tax-G exhibited comparable effects with lower cytotoxicity. These findings demonstrate that RMB and its active compounds protect muscle cells by regulating apoptosis and muscle metabolism, suggesting their potential as safe and functional natural materials for the prevention of sarcopenia.

## 1. Introduction

Skeletal muscle accounts for more than 50% of the body’s protein mass as voluntary muscle and plays a pivotal role not only in movement and posture maintenance but also in the regulation of systemic metabolic homeostasis through thermoregulation and the secretion of myokines [1,2,3]. Skeletal muscle is subject to atrophy with aging, leading to sarcopenia, which results in decreased muscle strength and physical function, increased risk of falls and fractures, and ultimately loss of independent living ability [4]. Furthermore, sarcopenia has been reported to be closely associated with severe chronic diseases such as obesity, diabetes, hyperglycemia, non-alcoholic fatty liver disease (NAFLD), cardiovascular and kidney diseases, and cancer [2,5]. In addition, with the acceleration of global population aging, the number of individuals affected by sarcopenia worldwide is projected to reach approximately 500 million by 2050 [6]. According to data reported by the Asian Working Group for Sarcopenia (AWGS) in 2020, the prevalence of sarcopenia among older adults in Asian populations was reported to be as high as 25.7% [7]. Consequently, the socioeconomic burden associated with sarcopenia is expected to increase substantially. Studies published in 2019 estimated that the annual additional healthcare and social care costs attributable to muscle weakness in the United Kingdom amounted to approximately £2.5 billion [8]. Similarly, in the United States, the total annual hospitalization costs for patients with sarcopenia were reported to reach approximately $40.4 billion [9]. Accordingly, sarcopenia is no longer regarded as a normal consequence of aging but has been assigned a diagnostic code in the International Classification of Diseases (ICD) by the World Health Organization (WHO) [10]. Furthermore, sarcopenia is now recognized as a disease in multiple countries, including the United States, Europe, Japan, and South Korea [5,7,9,11].

For the prevention and treatment of sarcopenia, it is necessary to understand the pathological mechanisms that induce muscle atrophy and to develop candidate substances that can fundamentally regulate them. Sarcopenia occurs due to multifactorial causes such as physical inactivity, oxidative stress, chronic inflammation, and hormonal changes, but the core pathogenesis is known to involve apoptosis in muscle cells and the collapse of muscle protein homeostasis caused by the imbalance between protein synthesis and degradation [5,6,12,13]. In particular, reactive oxygen species (ROS), which increase with aging and excessive exercise, impair mitochondrial function and induce apoptotic pathways, including alterations in the Bax/Bcl-2 ratio, caspase activation, and PARP cleavage [6,12,13]. Hydrogen peroxide (H_2_O_2_), a representative ROS, is widely used in vitro models to induce apoptosis via oxidative stress. On the other hand, dexamethasone (DEX), a synthetic glucocorticoid, regulates IGF-1/Akt/mTOR and FoxO signaling pathways, thereby suppressing the expression of muscle synthesis-related transcription factors MyoD and Myogenin, while upregulating muscle degradation factors Atrogin-1 and MuRF1, resulting in myotube atrophy [3,14,15,16]. Therefore, H_2_O_2_-induced apoptosis and DEX-induced muscle atrophy models in C2C12 cells have been widely utilized as representative platforms for investigating the pathological mechanisms of sarcopenia and to evaluate the efficacy of potential therapeutic candidates [16,17,18,19,20].

Recently, natural bioactive compounds have attracted attention for their potential to contribute to the prevention and treatment of sarcopenia through biological mechanisms such as antioxidant, anti-inflammatory, and anti-apoptotic activities [17,18,19,20,21,22]. Among them, *Rhododendron mucronulatum* is a native plant distributed throughout East Asia, including Korea, China, Japan, Mongolia, and Russia, and has traditionally been used for the treatment of respiratory diseases, pain, bleeding, and inflammation [23,24]. Recent studies have reported that extracts from the roots and bark of *R. mucronulatum* exhibit diverse activities, including antioxidant, anti-inflammatory, anticancer, immunomodulatory, and tyrosinase-inhibitory effects [24,25,26,27]. In particular, *R. mucronulatum* root extract has been shown to enhance cell viability in C2C12 oxidative stress models, suggesting its potential as a sarcopenia improving material [28].

Phytochemical profiling of *R. mucronulatum* has revealed that it contains abundant flavonoids, among which taxifolin-3-O-arabinopyranoside (Tax-G) is recognized as a chemotaxonomic marker of the genus *Rhododendron* [23,29,30]. Tax-G has been reported to possess various bioactivities such as antioxidant and anti-apoptotic effects, as well as skin-whitening, anti-wrinkle, and anti-hair loss properties [31,32,33]. The aglycone form of Tax-G, taxifolin (Tax-A), is known for its strong antioxidant activity [34,35] and muscle-protective properties, including inhibition of oxidative stress-induced muscle atrophy in rat [36], improvement of exercise performance in mouse [37], and suppression of apoptosis [34,35,38]. However, because Tax-A is present only in trace amounts in nature, an eco-friendly and safe enzymatic hydrolysis method for converting Tax-G into Tax-A has been developed [39,40].

Nevertheless, previous studies have several limitations. First, research on *R. mucronulatum* extracts has predominantly focused on the roots, which poses ecological problems during harvesting. Root harvesting causes plant death, soil structure destruction, loss of nutrients and carbon capital, and threats to biodiversity; therefore, avoidance of root damage is recommended in the harvesting of medicinal plants [41,42,43]. Moreover, roots are tightly bound to the soil and need extensive washing and preprocessing. In contrast, branches are easier to harvest, require minimal preparation, and provide a more environmentally sustainable resource, as they regenerate readily. Furthermore, recent research has confirmed that branches also contain a high content of Tax-G [29,44]. Second, studies on *R. mucronulatum* extracts and their active compounds in muscle cells remain scarce, and comprehensive studies on biomarkers related to apoptosis and muscle atrophy are insufficient. Finally, comparative studies of extracts, Tax-G, and Tax-A under the same conditions are also lacking. Considering that flavonoid aglycones show strong bioactivity but low bioavailability, whereas glycosides show higher gastrointestinal stability and absorption, such comparative research is crucial [45,46,47].

Therefore, this study evaluated the effects of RMB, Tax-G, and Tax-A on H_2_O_2_-induced apoptosis model and DEX-induced muscle atrophy model in C2C12 cells. In particular, cell viability, myotube diameter, and various molecular and cellular biomarkers, including Bax/Bcl-2, caspase-3, PARP, MyoD, Myogenin, Atrogin-1, MuRF1, Akt, mTOR, and FoxO3, were analyzed to comprehensively elucidate the muscle-protective effects and mechanisms of these compounds. This study demonstrates the potential of branches as a sustainable natural resource, and provides academic and industrial significance by presenting RMB, Tax-G, and Tax-A as candidate natural therapeutics for the prevention and treatment of sarcopenia.

## 2. Results

### 2.1. Phytochemical Analysis

#### 2.1.1. Qualitative Analysis of RMB (TLC)

**Figure 1:**
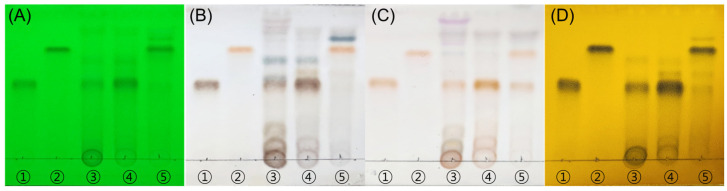
TLC analysis demonstrates the presence of Tax-G in RMB and the generation of Tax-A after enzymatic hydrolysis. (**A**) UV 254 nm, (**B**) 10% H_2_SO_4_, (**C**) *ρ*-anisaldehyde H_2_SO_4_, and (**D**) FeCl3. The eluent system employed was chloroform:methanol:water = 70:30:4 (*v*/*v*/*v*). ① Tax-A, ② Tax-G, ③ RMB, ④ RMB EA layer (RMB RF1), and ⑤ EA layer of enzyme-hydrolyzed RMB (RMB RF2). “RF” denotes “Rich Fraction”, indicating a fraction enriched with the target compound.

Thin-layer chromatography (TLC) analysis demonstrated the presence of taxifolin-3-O-arabinopyranoside (Tax-G) and taxifolin (Tax-A) in *Rhododendron mucronulatum* branch extract (RMB) and its derived fractions. By comparison with standards, Tax-G was identified in RMB and the ethyl acetate fraction of RMB (RMB RF1), whereas the ethyl acetate fraction of enzyme-hydrolyzed RMB (RMB RF2) showed the presence of Tax-A, indicating the conversion of Tax-G to its aglycone form (Figure 1).

#### 2.1.2. HPLC Quantitative Analysis and LC-MS/MS Analysis of RMB

As a result of standard validation for quantitative analysis, the calibration curve for taxifolin-3-O-arabinopyranoside (Tax-G) was calculated as y = 29308x + 63601 (R^2^ = 0.9997, LOD 2.94, LOQ 8.91), while that for taxifolin (Tax-A) was y = 53860x + 167612 (R^2^ = 0.9985, LOD 15.81, LOQ 47.92) (Figure S1).

**Figure 2:**
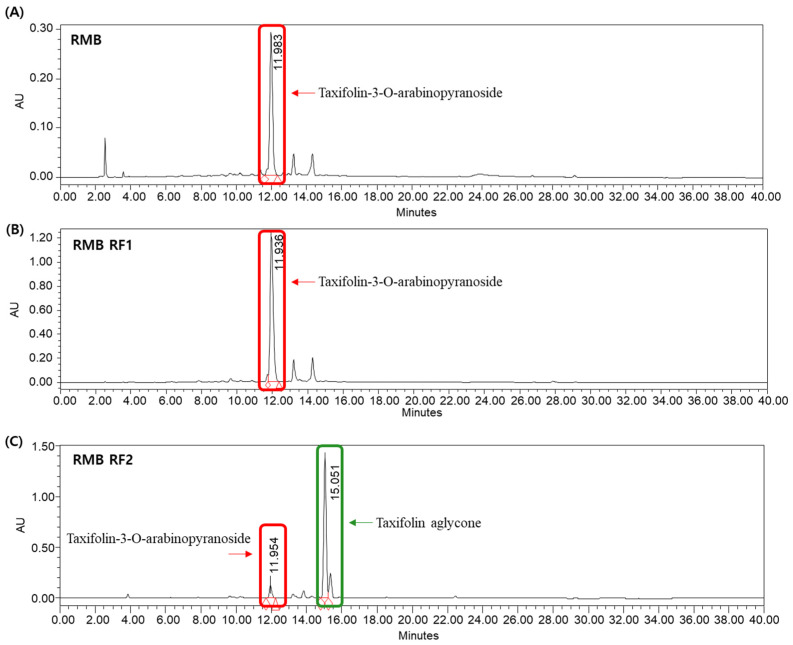
HPLC quantitative analysis showing that Tax-G is the major component of RMB, enriched in the ethyl acetate fraction and effectively converted to Tax-A after enzymatic hydrolysis. HPLC chromatogram of (**A**) *Rhododendron mucronulatum* branch extract powder (RMB; 1000 μg/mL, Tax-G: 107.47 ± 0.45 μg/mL), (**B**) EA layer RMB (RMB RF1; 1000 μg/mL, Tax-G: 500.00 ± 1.56 μg/mL), and (**C**) EA layer of enzyme-hydrolyzed RMB (RMB RF2; 1000 μg/mL, Tax-G: 41.81 ± 0.08 μg/mL, Tax-A: 249.95 ± 0.79 μg/mL).

The quantitative changes in Tax-G and Tax-A during the processing steps were monitored via HPLC (Figure 2). In RMB extract sample, the content of Tax-G was calculated to be 107.47 ± 0.45 μg/mL (n = 3), while Tax-A was not detected. In sample RMB RF1, Tax-G was further enriched to 500.00 ± 1.56 μg/mL (n = 3), while Tax-A remained undetectable. In sample RMB RF2, Tax-G content significantly decreased to 41.81 ± 0.08 μg/mL, while Tax-A content markedly increased to 249.95 ± 0.79 μg/mL (n = 3), indicating that the glycosylated form was effectively converted to the aglycone.

LC–MS/MS analysis in [M − H]^−^ negative ion mode further confirmed the identities of these compounds. A molecular ion peak at 435.2 m/z was detected in both the RMB and RF2 samples, corresponding to Tax-G. Notably, a peak at 303.0 m/z was observed in the RF2 sample, matching Tax-A (Figure S2) [48,49].

### 2.2. Chemical Separation and Identification

**Figure 3:**
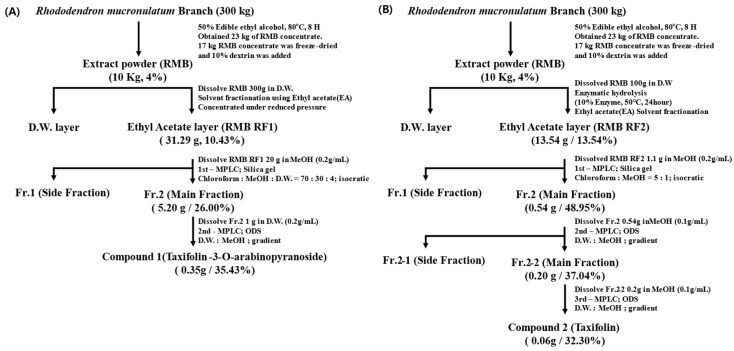
Flowchart of (**A**) taxifolin-3-O-arabinopyranoside isolation process and (**B**) taxifolin aglycone isolation process. The side fraction mainly consisted of other minor components. Fr. refers to the fraction, and D.W. refers to the distilled water.

Compound **1** and compound **2** were isolated and purified from RMB RF1 and RMB RF2, respectively. The purification flow charts for each compound are summarized in Figure 3.

Compound **1** and Compound **2** were qualitatively analyzed using TLC, HPLC, and LC-MS/MS (Figures S3–S5). The results confirmed that both isolated compounds correspond to their respective standard compounds. Notably, based on the LOD (5.02) of Tax-G in the HPLC analysis, Compound **1** exhibited a high purity of 98.21%, and Compound **2** showed a purity of 99.68%, demonstrating their suitability for subsequent biological experiments.

Subsequently, structural identification of compound **1** and compound **2** was performed through NMR analysis, and the results are as follows.

Compound **1**; a yellowish-white amorphous powder.

^1^H-NMR (700 MHz, MeOH-*d*_4_) δ(ppm): 6.97 (1H, d, *J* ≈ 2.0 Hz, H-2′), 6.89 (1H, dd, *J* ≈ 8.0, 2.0 Hz, H-6′), 6.85 (1H, d, *J* ≈ 8.0 Hz, H-5′), 5.92, 5.90 (each 1H, br s, H-6, H-8), 5.18 (1H, d, *J* ≈ 7.5 Hz, H-1″), 4.90–4.80 (1H, m, H-3), 4.75–4.60 (1H, m, H-2), 4.4–3.4 (5H, m, H-2″–H-5″, overlapped).

^13^C-NMR (176MHz, MeOH-*d*_4_) δ(ppm): 196.3 (C-4), 169.2 (C-7), 165.6 (C-5), 164.3 (C-9), 147.5 (C-4′), 146.6 (C-3′), 129.3 (C-1′), 120.9 (C-6′), 116.4 (C-5′), 116.0 (C-2′), 104.4 (C-1″), 102.5 (C-10), 97.6 (C-6), 96.5 (C-8), 83.9 (C-2), 77.7 (C-2″), 76.6 (C-3), 73.7 (C-3″), 71.3 (C-4″), 64.8 (C-5″).

Compound **2**; a yellowish-white amorphous powder.

^1^H-NMR (700 MHz, MeOH-*d*_4_) δ(ppm): 6.97 (1H, br s, H-2′), 6.85–6.80 (2H, m, H-5′, H-6′), 5.92 (1H, br s, H-6), 5.88 (1H, br s, H-8), 4.92 (1H, d, *J* = 11.5 Hz, H-2), 4.51 (1H, d, *J* = 11.5 Hz, H-3).

^13^C-NMR (176MHz, MeOH-*d*_4_) δ(ppm): 198.5 (C-4), 168.8 (C-7), 165.4 (C-5), 164.6 (C-9), 147.3 (C-4′), 146.4 (C-3′), 130.0 (C-1′), 121.1 (C-6′), 116.3 (C-5′), 116.1 (C-2′), 102.0 (C-10), 97.5 (C-6), 96.4 (C-8), 85.2 (C-2), 73.8 (C-3).

Compound **1** (Tax-G) and compound **2** (Tax-A) were consistent with the spectral data reported in previous studies [31,40,50], thereby confirming that the two compounds isolated and purified from RMB were taxifolin-3-O-arabinopyranoside and taxifolin aglycone, respectively.

### 2.3. Impact of Rhododendron mucronulatum Branch Extract (RMB), Taxifolin-3-O-Arabinopyranoside (Tax-G) and Taxifolin (Tax-A) the Viability of C2C12 Myoblasts

#### 2.3.1. Measurement of Cell Viability Under Normal Conditions

**Figure 4:**
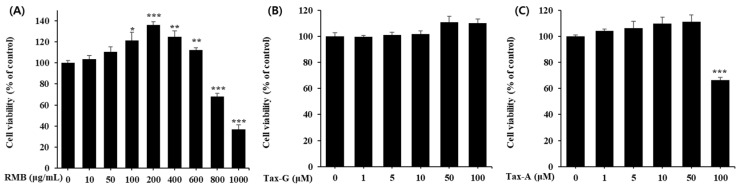
Cytotoxicity of (**A**) *Rhododendron mucronulatum* branch extract (RMB), (**B**) Taxifolin-3-O-arabinopyranoside (Tax-G) and (**C**) Taxifolin (Tax-A) on C2C12 myoblasts. Cell viability was calculated as described in Materials and Methods. Values are expressed as the mean ± S.E.M. (n = 5). * *p* < 0.05, ** *p* < 0.01, and *** *p* < 0.001 indicate significantly different from the 0 μg/mL group.

To investigate the cytotoxicity of the three test substances (RMB, Tax-G, Tax-A) on C2C12 myoblasts, cells were treated with various concentrations (RMB, 0–1000 μg/mL; Tax-G and Tax-A, 0–100 μM) for 48 h, followed by the MTT assay. Compared with the control group, RMB treatment significantly increased cell viability in the concentration range of 100–600 μg/mL, showing the highest viability at 200 μg/mL, whereas viability significantly decreased at concentrations of 800 and 1000 μg/mL (Figure 4A). Tax-G treatment did not affect cell viability at any concentration (Figure 4B). In contrast, Tax-A treatment reduced cell viability by 33.6% at 100 μM compared with the control group (0 μg/mL) (Figure 4C). Based on these results, the highest treatment concentrations of RMB, Tax-G, and Tax-A were set at 200 μg/mL, 100 μM, and 50 μM, respectively, for subsequent experiments.

#### 2.3.2. Impact of H_2_O_2_-Induced Myoblast Damage

**Figure 5:**
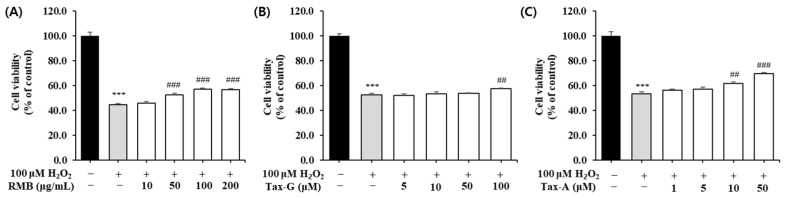
Protective effect of (**A**) *Rhododendron mucronulatum* branch extract (RMB), (**B**) Taxifolin-3-O-arabinopyranoside (Tax-G) and (**C**) Taxifolin (Tax-A) on cell viability in H_2_O_2_-treated C2C12 myoblasts. Values are expressed as the mean ± S.E.M. (n = 4). *** *p* < 0.001 indicates significantly different from the [H_2_O_2_ (−)/Treatment (−)] group. ## *p* < 0.01 and ### *p* < 0.001 indicate significantly different from the [H_2_O_2_ (+)/Treatment (−)] group. Treatment refers to the administration of RMB, Tax-G, or Tax-A.

To investigate the protective effects of the three test substances against oxidative stress-induced muscle cell damage, oxidative stress was induced in C2C12 myoblasts by treatment with 100 μM H_2_O_2_. The cells were then treated with the three test substances at various concentrations and cultured for 48 h, after which cell viability was measured. Compared with the control group, the H_2_O_2_-treated group showed a significant decrease in cell viability (Figure 5). Treatment with RMB (10, 50, 100, and 200 μg/mL) significantly increased cell viability compared with the H_2_O_2_-treated group at concentrations above 50 μg/mL, with a maximum increase of 28.1%, reaching 57.4 ± 0.7% (Figure 5A). Treatment with Tax-G (5, 10, 50, and 100 μM) significantly increased cell viability at 100 μM, showing an increase of approximately 9.7% compared with the H_2_O_2_-treated group, reaching 57.7 ± 0.3% (Figure 5B). Treatment with Tax-A (1, 5, 10, and 50 μM) significantly increased cell viability at concentrations above 10 μM, with the maximum concentration of 50 μM showing an increase of approximately 30.8% compared with the H_2_O_2_-treated group, reaching 69.8 ± 0.7% (Figure 5C).

#### 2.3.3. Impact of Dexamethasone-Induced Myotube Damage

**Figure 6:**
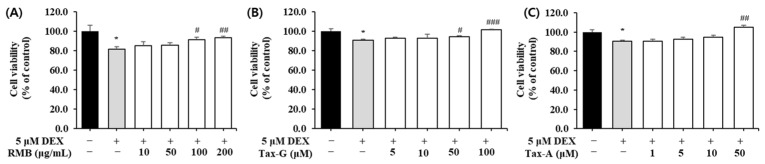
Protective effect of (**A**) *Rhododendron mucronulatum* branch extract (RMB), (**B**) Taxifolin-3-O-arabinopyranoside (Tax-G) and (**C**) Taxifolin (Tax-A) on cell viability in dexamethasone-induced muscle atrophy in C2C12 myoblasts. Values are expressed as the mean ± S.E.M. (n = 4). * *p* < 0.05 indicates significantly different from the [DEX (−)/Treatment (−)] group. # *p* < 0.05, ## *p* < 0.01 and ### *p* < 0.001 indicate significantly different from the [DEX (+)/Treatment (−)] group. Treatment refers to the administration of RMB, Tax-G, or Tax-A.

To investigate the effects of the three test substances on dexamethasone (DEX)-induced muscle cell damage, each substance was applied to a C2C12 muscle atrophy model induced with 5 μM DEX, and the cell viability of myotubes was measured. Compared with the control group, the cell viability of the DEX-treated group decreased to as low as 81.8 ± 2.4%. RMB treatment significantly increased cell viability at concentrations of 100 and 200 μg/mL (Figure 6A), Tax-G treatment at concentrations of 50 and 100 μM (Figure 6B), and Tax-A treatment at a concentration of 50 μM (Figure 6C), compared with the DEX-treated group.

### 2.4. Effects of Rhododendron mucronulatum Branch Extract (RMB), Taxifolin-3-O-Arabinopyranoside (Tax-G) and Taxifolin (Tax-A) on Muscle Apoptosis Biomarkers

#### 2.4.1. Effects on H_2_O_2_-Induced Apoptosis in Myoblasts

**Figure 7:**
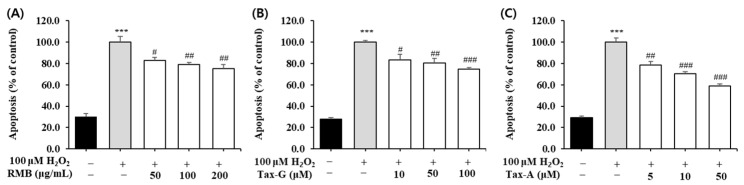
Protective effect of (**A**) *Rhododendron mucronulatum* branch extract (RMB), (**B**) Taxifolin-3-O-arabinopyranoside (Tax-G) and (**C**) Taxifolin (Tax-A) on apoptosis in H_2_O_2_-treated C2C12 myoblasts. Values are expressed as the mean ± S.E.M. (n = 4). *** *p* < 0.001 indicates significantly different from the [H_2_O_2_ (−)/Treatment (−)] group. # *p* < 0.05, ## *p* < 0.01, and ### *p* < 0.001 indicate significantly different from the [H_2_O_2_ (+)/Treatment (−)] group. Treatment refers to the administration of RMB, Tax-G, or Tax-A.

To evaluate the effects of RMB, Tax-G, and Tax-A on oxidative stress-induced apoptosis, DNA fragmentation was quantified using a Cellular DNA Fragmentation ELISA kit. The H_2_O_2_-treated group showed a significant increase in apoptosis compared with the untreated control group (Figure 7). RMB treatment significantly reduced apoptosis starting at 50 μg/mL, and at the highest concentration of 200 μg/mL, apoptosis decreased by 24.7% compared with the H_2_O_2_-treated group (Figure 7A). Tax-G treatment significantly reduced apoptosis starting at 10 μM, and at the highest concentration of 100 μM, apoptosis decreased by 25.5% compared with the H_2_O_2_-treated group (Figure 7B). Tax-A treatment significantly reduced apoptosis starting at 5 μM, and at the highest concentration of 50 μM, apoptosis decreased by 41.1% compared with the H_2_O_2_-treated group (Figure 7C).

#### 2.4.2. Effects of RMB, Tax-G and Tax-A on Bax, Bcl-2, Cleaved Caspase-3, and Cleaved PARP Protein Expression

**Figure 8:**
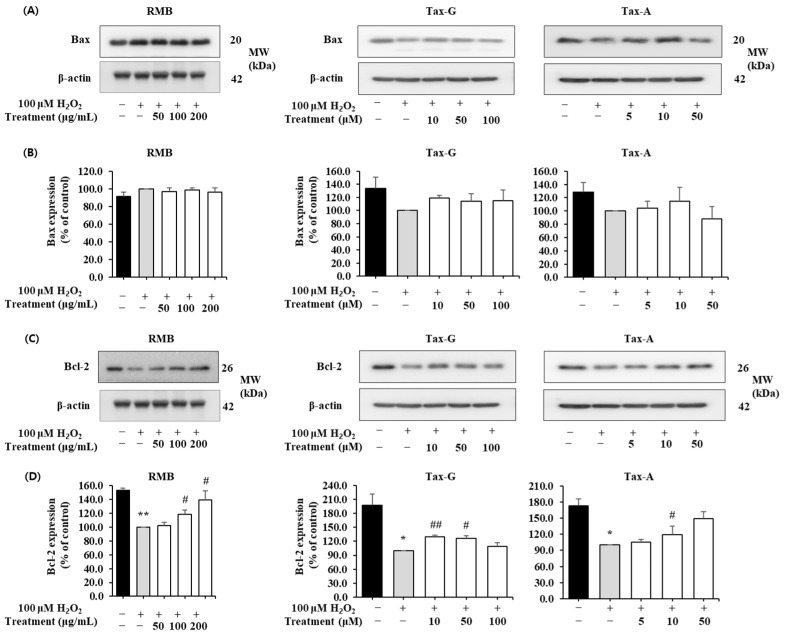
Anti-apoptotic effects of *Rhododendron mucronulatum* branch extract (RMB), Taxifolin-3-O-arabinopyranoside (Tax-G) and Taxifolin (Tax-A) on H_2_O_2_-induced oxidative damage in C2C12 myoblasts. Western blotting was used to analyze the levels of (**A**,**B**) Bax, (**C**,**D**) Bcl-2, and β-actin, as described in Materials and Methods. Their relative protein expressions were normalized to β-actin. TM indicates treatment (RMB, Tax-G, Tax-A). Values are expressed as the mean ± S.E.M. (n = 3). * *p* < 0.05, ** *p* < 0.01 indicate significantly different from the [H_2_O_2_ (−)/Treatment (−)] group. # *p* < 0.05, ## *p* < 0.01 indicate significantly different from the [H_2_O_2_ (+)/Treatment (−)] groups. Treatment refers to the administration of RMB, Tax-G, or Tax-A.

**Figure 9:**
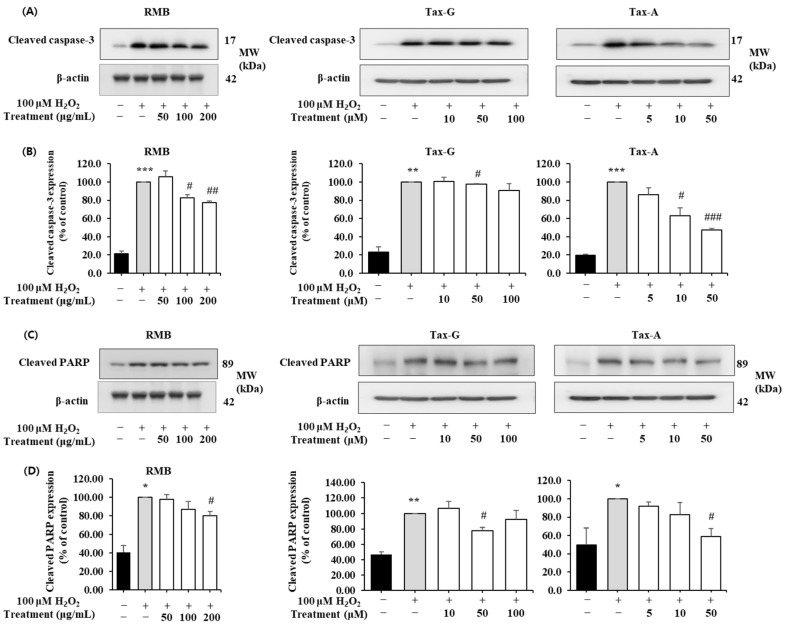
Anti-apoptotic effects of *Rhododendron mucronulatum* branch extract (RMB), Taxifolin-3-O-arabinopyranoside (Tax-G) and Taxifolin (Tax-A) on H_2_O_2_-induced oxidative damage in C2C12 myoblasts. Western blotting was used to analyze the levels of (**A**,**B**) Cleaved caspase-3, (**C**,**D**) Cleaved PARP, and β-actin, as described in Materials and Methods. Their relative protein expressions were normalized to β-actin. TM indicates treatment (RMB, Tax-G, Tax-A). Values are expressed as the mean ± S.E.M. (n = 3). * *p* < 0.05, ** *p* < 0.01, and *** *p* < 0.001 indicate significantly different from the [H_2_O_2_ (−)/Treatment (−)] group. # *p* < 0.05, ## *p* < 0.01, and ### *p* < 0.001 indicate significantly different from the [H_2_O_2_ (+)/Treatment (−)] groups. Treatment refers to the administration of RMB, Tax-G, or Tax-A.

In the H_2_O_2_-induced apoptosis model, the effects of RMB, Tax-G, and Tax-A treatments on the expression of Bax, Bcl-2, cleaved caspase-3, and cleaved PARP proteins were analyzed, and the results are shown in Figure 8 and Figure 9. The expression of Bax did not show significant differences among the control group, the H_2_O_2_-treated group, and the groups treated with the test substances (Figure 8A,B). In contrast, the expression of Bcl-2 was significantly decreased in the H_2_O_2_-treated group compared with the control group. This decrease was significantly restored by treatment with RMB (100 and 200 μg/mL), Tax-G (10 and 50 μM), and Tax-A (50 μM) (Figure 8D). In particular, Bcl-2 expression in the groups treated with RMB (200 μg/mL), Tax-G (10 μM), and Tax-A (50 μM) increased by 38.8%, 30.1%, and 48.7%, respectively, compared with the H_2_O_2_-treated group.

The expression of cleaved caspase-3 and cleaved PARP was significantly increased in the H_2_O_2_-treated group compared with the control group. However, these increases were significantly suppressed by treatment with RMB (100 and 200 μg/mL), Tax-G (10 and 50 μM), and Tax-A (50 μM) (Figure 9A–D). In particular, compared with the H_2_O_2_-treated group, the expression of cleaved caspase-3 decreased by 32.2% in the RMB 200 μg/mL group, 28.3% in the Tax-G 50 μM group, and 41.6% in the Tax-A 50 μM group. Similarly, the expression of cleaved PARP significantly decreased by 30.2% (RMB 200 μg/mL), 27.6% (Tax-G 50 μM), and 39.9% (Tax-A 50 μM), respectively, compared with the H_2_O_2_-treated group.

### 2.5. Effects of Rhododendron mucronulatum Branch Extract (RMB), Taxifolin-3-O-Arabinopyranoside (Tax-G) and Taxifolin (Tax-A) on Muscle-Synthesis- and Muscle-Degradation-Related Proteins and Gene Expression

#### 2.5.1. Dexamethasone-Induced Myotube Atrophy

**Figure 10:**
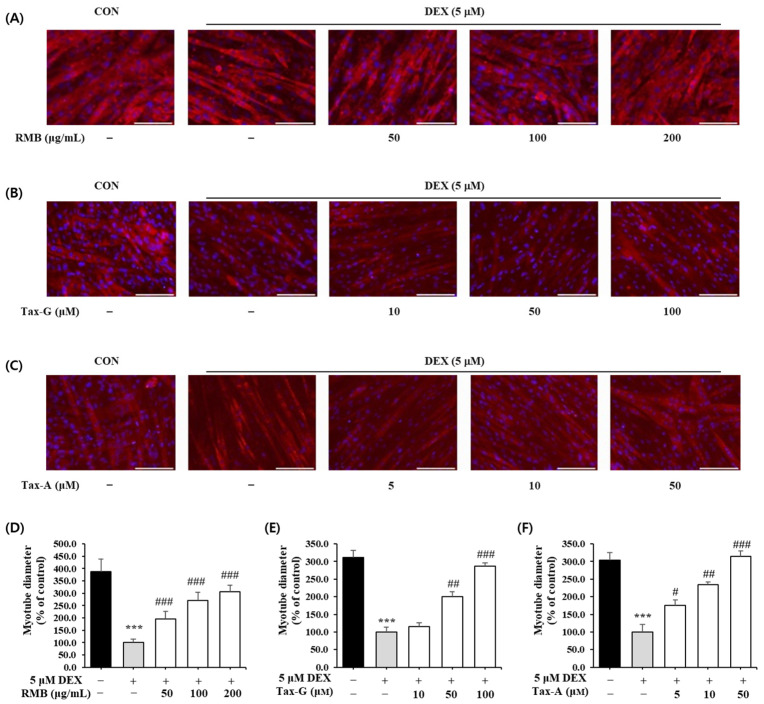
Effect of (**A**,**D**) *Rhododendron mucronulatum* branch extract (RMB), (**B**,**E**) Taxifolin-3-O-arabinopyranoside (Tax-G) and (**C**,**F**) Taxifolin (Tax-A) on dexamethasone-induced muscle atrophy in C2C12 myotubes. Values are expressed as the mean ± S.E.M. (n = 5). *** *p* < 0.001 indicates significantly different from the [DEX (−)/Treatment (−)] group. # *p* < 0.05, ## *p* < 0.01, and ### *p* < 0.001 indicate significantly different from the [DEX (+)/Treatment (−)] group. Treatment refers to the administration of RMB, Tax-G, or Tax-A at the indicated concentrations.

The effects of the three test substances on dexamethasone (DEX)-induced muscle atrophy were evaluated by measuring the diameter of myotubes. Treatment with 5 μM DEX significantly reduced myotube diameter by approximately 70.3% compared with the control group (Figure 10). In contrast, treatment with RMB, Tax-G, and Tax-A significantly increased myotube diameters in a concentration-dependent manner compared with the DEX-treated group, and at the highest concentrations, the diameters were restored to control levels. At their highest concentrations, RMB, Tax-G, and Tax-A increased myotube diameters by 206.3%, 186.1%, and 215.0%, respectively, compared with the DEX-treated group (Figure 10).

#### 2.5.2. Expression of Atrogin-1, MuRF1, Myogenin, and MyoD Protein in Dexamethasone-Treated C2C12 Myotubes

**Figure 11:**
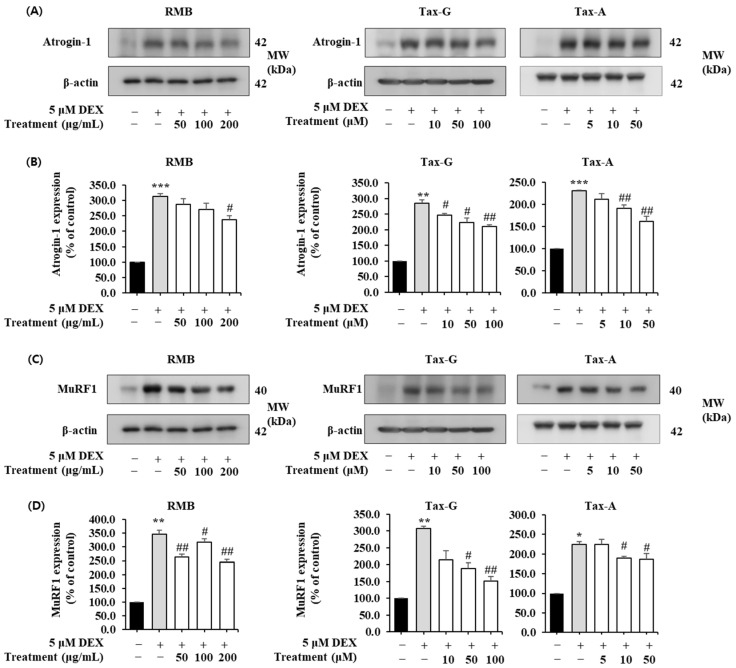
Effect of *Rhododendron mucronulatum* branch extract (RMB), Taxifolin-3-O-arabinopyranoside (Tax-G) and Taxifolin (Tax-A) on the muscle degradation-related protein expression of (**A**,**B**) Atrogin-1, (**C**,**D**) MuRF1 in dexamethasone-treated C2C12 myotubes. Their relative protein expressions were normalized to β-actin. Values are expressed as the mean ± S.E.M. (n = 3). * *p* < 0.05, ** *p* < 0.01, and *** *p* < 0.001 indicate significantly different from the DEX (−)/Treatment (−)] group. # *p* < 0.05, ## *p* < 0.01 indicate significantly different from the [DEX (+)/Treatment (−)] group. Treatment refers to the administration of RMB, Tax-G, or Tax-A. For Tax-A and Tax-G.

Protein expression of the muscle degradation-related factors Atrogin-1 and MuRF1 was significantly increased in the DEX-treated group compared with the control group (Figure 11). In the case of Atrogin-1, treatment with the test substances reduced its expression in a concentration-dependent manner; RMB significantly reduced expression at 200 μg/mL, and Tax-G and Tax-A significantly reduced expression at concentrations of 10 μM or higher (Figure 11A,B). At the highest treatment concentrations of each group, Atrogin-1 expression decreased by 23.97%, 26.23%, and 29.86% with RMB, Tax-G, and Tax-A treatment, respectively, compared with the DEX-treated group. Meanwhile, MuRF expression was significantly reduced at all concentrations of RMB, at 50 and 100 μM of Tax-G, and at 10 and 50 μM of Tax-A (Figure 11C,D). At the highest treatment concentrations, MuRF expression decreased by 34.95%, 46.46%, and 16.03% with RMB, Tax-G, and Tax-A treatment, respectively, compared with the DEX-treated group.

**Figure 12:**
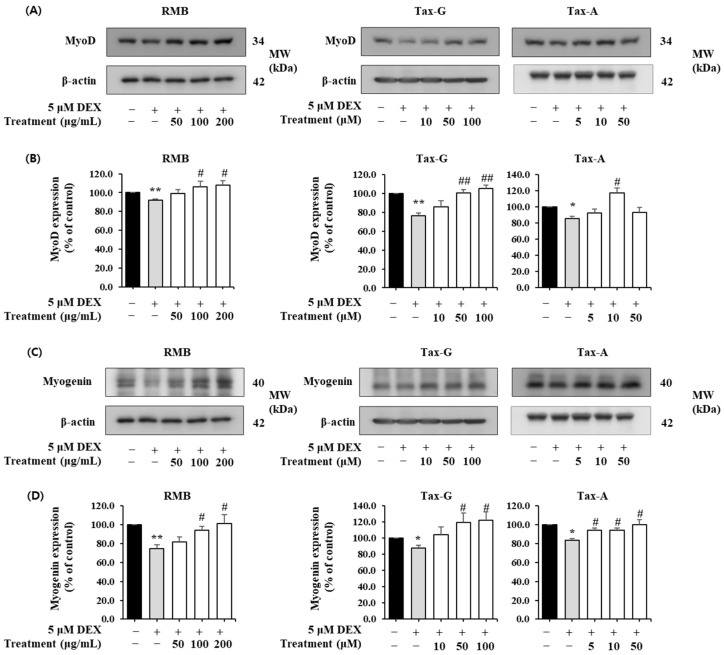
Effect of *Rhododendron mucronulatum* branch extract (RMB), Taxifolin-3-O-arabinopyranoside (Tax-G), and Taxifolin (Tax-A) on the muscle degradation-related protein expression of (**A**,**B**) MyoD and (**C**,**D**) Myogenin in dexamethasone-treated C2C12 myotubes. Their relative protein expressions were normalized to β-actin. Values are expressed as the mean ± S.E.M. (n = 3). * *p* < 0.05 and ** *p* < 0.01 indicate significantly different from the [DEX (−)/Treatment (−)] group. # *p* < 0.05 and ## *p* < 0.01 indicate significantly different from the [DEX (+)/Treatment (−)] group. Treatment refers to the administration of RMB, Tax-G, or Tax-A.

In contrast, the expression of the muscle synthesis-related proteins MyoD and Myogenin was significantly decreased in the DEX-treated group compared with the control group (Figure 12). MyoD expression was significantly increased at 100 and 200 μg/mL of RMB, at 50 and 100 μM of Tax-G, and at 10 μM of Tax-A (Figure 12A,B). Compared with the DEX-treated group, MyoD expression increased by 17.66% with 200 μg/mL RMB, 38.30% with 100 μM Tax-G, and 37.93% with 10 μM Tax-A. Myogenin expression was significantly increased at 100 and 200 μg/mL of RMB, at 50 and 100 μM of Tax-G, and at all treatment concentrations of Tax-A (Figure 12C,D). At the highest treatment concentrations, Myogenin expression increased by 35.05%, 39.67%, and 20.29% with RMB, Tax-G, and Tax-A, respectively, compared with the DEX-treated group.

#### 2.5.3. Effects on Muscle-Degradation- and Synthesis-Related Gene Expression

To evaluate the effects of the test substances on muscle degradation and synthesis mechanisms, the mRNA expression was also analyzed. Compared with the control group, the expression of Atrogin-1 and MuRF1 mRNA was significantly increased in the DEX-treated group, whereas the expression of MyoD1 and Myogenin mRNA was significantly decreased (Table 1, Table 2 and Table 3).

Atrogin-1 mRNA expression was significantly decreased at 100 and 200 μg/mL of RMB, at 100 μM of Tax-G, and at all treatment concentrations of Tax-A. Compared with the DEX-treated group, Atrogin-1 expression decreased by 49.41% with 100 μg/mL RMB, 49.13% with 100 μM Tax-G, and 40.62% with 50 μM Tax-A. MuRF1 mRNA expression was significantly decreased at 100 and 200 μg/mL of RMB, at 50 and 100 μM of Tax-G, and at 10 and 50 μM of Tax-A, with maximum reductions of 64.38%, 44.39%, and 37.35%, respectively, compared with the DEX-treated group.

MyoD1 mRNA expression was significantly increased at 100 and 200 μg/mL of RMB, at 50 and 100 μg/mL of Tax-G, and at 10 and 50 μg/mL of Tax-A. Compared with the DEX-treated group, MyoD1 expression increased by 94.11% with 100 μg/mL RMB, 205.36% with 100 μM Tax-G, and 117.62% with 50 μM Tax-A. Myogenin mRNA expression was significantly increased at 200 μg/mL of RMB and at all concentrations of Tax-G and Tax-A, with maximum increases of 70.43%, 148.93%, and 97.65%, respectively, compared with the DEX-treated group.

#### 2.5.4. Akt/mTOR/FoxO-Signaling-Pathway-Related Muscle Protein Expression Investigation

**Figure 13:**
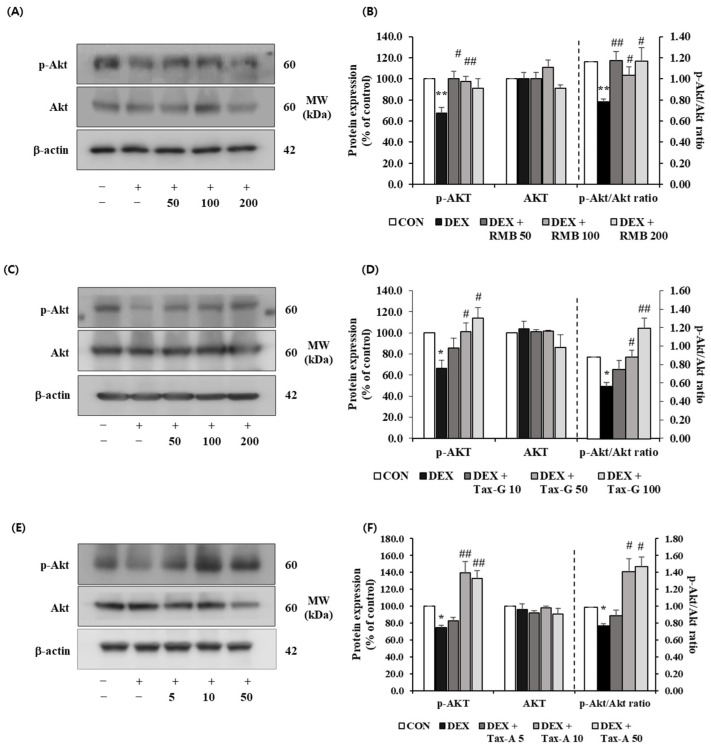
Effect of (**A**,**B**) *Rhododendron mucronulatum* branch extract (RMB), (**C**,**D**) Taxifolin-3-O-arabinopyranoside (Tax-G), and (**E**,**F**) Taxifolin (Tax-A) on the protein expression of phospho-Akt and Akt in DEX-treated C2C12 myotubes. Protein expression was assessed via Western blotting, with β-actin as the normalization standard, and expressed relative to the CON group. Values are reported as the mean ± S.E.M. (n = 3). * *p* < 0.05, ** *p* < 0.01 indicate significantly different from the [DEX (−)/Treatment (−)] group. # *p* < 0.05, ## *p* < 0.01 indicate significantly different from the [DEX (+)/Treatment (−)] groups. Treatment refers to the administration of RMB, Tax-G, or Tax-A.

The expression of p-Akt was significantly decreased in the DEX-treated group compared with the control group, whereas treatment with RMB (50 and 100 μg/mL), Tax-G (50 and 100 μM), and Tax-A (10 and 50 μM) significantly restored the reduced expression of p-Akt (Figure 13). The expression of Akt did not differ significantly among the control, DEX-treated groups and any treatment groups. The p-Akt/Akt ratio was significantly decreased in the DEX-treated group compared with the control group. This decrease was significantly reversed at all treatment concentrations of RMB, at 50 and 100 μM of Tax-G, and at 10 and 50 μM of Tax-A (Figure 13).

**Figure 14:**
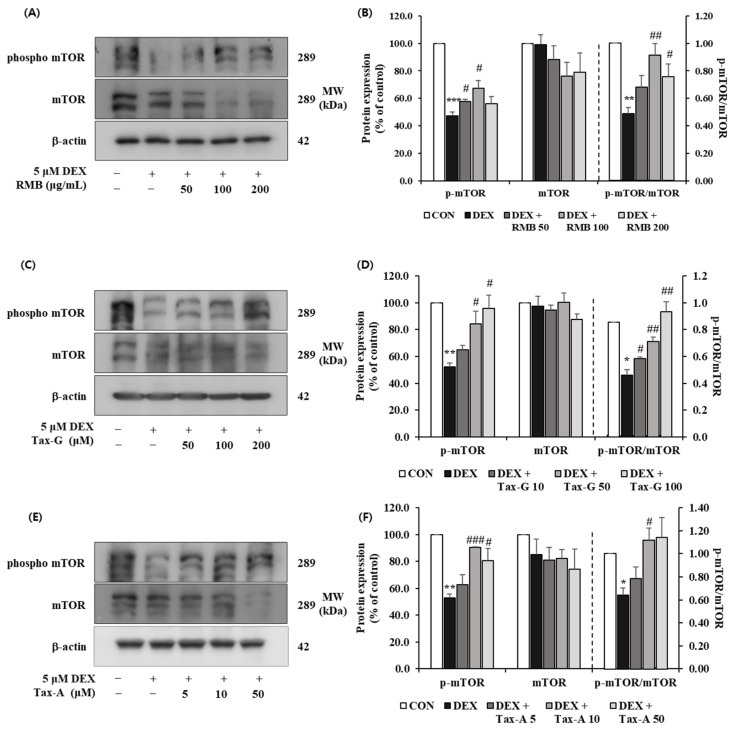
Effect of (**A**,**B**) *Rhododendron mucronulatum* branch extract (RMB), (**C**,**D**) Taxifolin-3-O-arabinopyranoside (Tax-G), and (**E**,**F**) Taxifolin (Tax-A) on the protein expression of phospho-mTOR and mTOR in DEX-treated C2C12 myotubes. Protein expression was assessed via Western blotting, with β-actin as the normalization standard, and expressed relative to the CON group. Values are reported as the mean ± S.E.M. (n = 3). * *p* < 0.05, ** *p* < 0.01, and *** *p* < 0.001 indicate significantly different from the [DEX (−)/Treatment (−)] group. # *p* < 0.05, ## *p* < 0.01, and ### *p* < 0.001 indicate significantly different from the [DEX (+)/Treatment (−)] groups. Treatment refers to the administration of RMB, Tax-G, or Tax-A.

The expression of p-mTOR was significantly reduced in the DEX-treated group compared with the control group, whereas treatment with RMB (50 and 100 μg/mL), Tax-G (50 and 100 μM), and Tax-A (10 and 50 μM) significantly restored the expression of p-mTOR. The expression of mTOR did not differ significantly among groups. The p-mTOR/mTOR ratio was significantly decreased in the DEX-treated group compared with the control group. This decrease was significantly reversed at 100 and 200 μg/mL of RMB, at all treatment concentrations of Tax-G, and at 10 μM of Tax-A (Figure 14).

**Figure 15:**
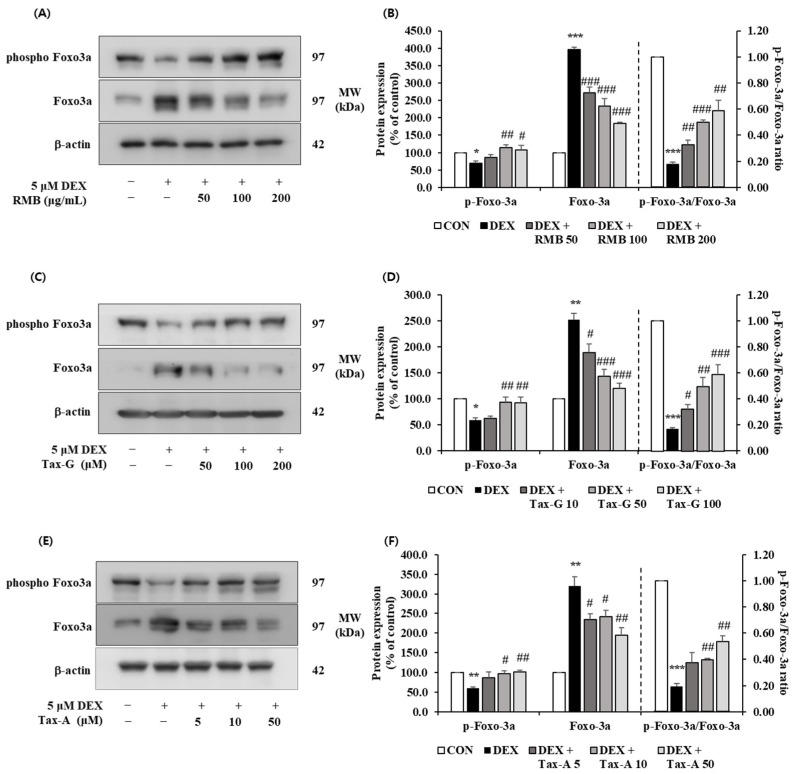
Effect of (**A**,**B**) *Rhododendron mucronulatum* branch extract (RMB), (**C**,**D**) Taxifolin-3-O-arabinopyranoside (Tax-G), and (**E**,**F**) Taxifolin (Tax-A) on the protein expression of phospho-FoxO3a and FoxO3a in DEX-treated C2C12 myotubes. Protein expression was assessed via Western blotting, with β-actin as the normalization standard, and expressed relative to the CON group. Values are reported as the mean ± S.E.M. (n = 3). * *p* < 0.05, ** *p* < 0.01, and *** *p* < 0.001 indicate significantly different from the [DEX (−)/Treatment (−)] group. # *p* < 0.05, ## *p* < 0.01, and ### *p* < 0.001 indicate significantly different from the [DEX (+)/Treatment (−)] groups. Treatment refers to the administration of RMB, Tax-G, or Tax-A.

The expression of p-FoxO3a was significantly reduced in the DEX-treated group compared with the control group, and the reduced expression of p-FoxO3a by DEX treatment was significantly increased at 100 and 200 μg/mL of RMB, at 50 and 100 μM of Tax-G, and at 10 and 50 μM of Tax-A. The expression of FoxO3a was significantly increased in the DEX-treated group compared with the control group, and the increased expression of FoxO3a by DEX treatment was significantly decreased at all concentrations of RMB, Tax-G, and Tax-A. The p-FoxO3a/FoxO3a ratio was significantly decreased in the DEX-treated group compared with the control group, and this decrease was significantly reversed at all concentrations of RMB and Tax-G, and at 10 and 50 μM of Tax-A (Figure 15).

## 3. Discussion

Apoptosis and the imbalance of protein metabolism in muscle cells are recognized as key pathological mechanisms involved in the onset and progression of sarcopenia [6,51]. This study aimed to evaluate the potential of *Rhododendron mucronulatum* branch extract (RMB) and its active compounds, taxifolin-3-O-arabinopyranoside (Tax-G) and taxifolin (Tax-A), in modulating these mechanisms. In this study, an apoptosis model induced by H_2_O_2_ and a muscle atrophy model induced by dexamethasone (DEX) were established in C2C12 cells. Various molecular and cellular biomarkers, such as protein expression and myotube diameter changes, were analyzed to comprehensively assess the protective effects of RMB, Tax-G, and Tax-A on muscle cells, and to compare their relative activities.

Prior to the in vitro experiments, phytochemical analysis revealed that RMB extracted at pilot scale contained a high content of Tax-G. This demonstrated the efficiency of the pilot-scale extraction process and, furthermore, strengthened its industrial applicability when considering the sustainability of raw material acquisition. In addition, it was confirmed that RMB could be enzymatically hydrolyzed to produce a high content of Tax-A. This suggests the possibility of applying this process as an eco-friendly and safe method to produce the high value-added compound Tax-A. These findings provided both a chemical basis for the in vitro experiments and insights into the industrial applicability of RMB.

Prior to evaluating muscle loss and muscle atrophy models, the concentration-dependent effects of the test substances on cell viability under normal conditions were assessed to exclude cytotoxicity and establish a safe concentration range. Analysis of cell viability revealed significant cytotoxicity at concentrations of RMB at 800 μg/mL and above, as well as Tax-A at 100 μM. Such reductions in cell viability are commonly observed as concentration-dependent responses in in vitro cell-based experiments and may be associated with non-specific cellular stress or increased metabolic burden under high-concentration conditions [52,53,54,55,56]. In addition, Tax-G did not exhibit increased cytotoxicity compared with its aglycone form under the tested conditions, which may indicate differences in cellular behavior between the glycoside and aglycone forms. Meanwhile, RMB demonstrated a tendency to increase cell viability at concentrations up to 200 μg/mL and did not exhibit significant cytotoxicity at concentrations up to 600 μg/mL. This finding may result from various factors, including enhanced cell proliferation, alterations in metabolic activity, or assay-dependent signal variations, and therefore, the underlying mechanism cannot be conclusively determined based solely on the results of this experiment. Furthermore, treatment with RMB, Tax-G, and Tax-A significantly restored muscle cell damage induced by H_2_O_2_ and DEX. This suggests the potential of each material for antioxidant, anti-apoptotic, and muscle-protective effects, which were verified through biomarkers of apoptosis and protein synthesis–degradation.

In the oxidative stress model, the H_2_O_2_-treated group showed a marked increase in apoptosis. Western blot analysis showed that H_2_O_2_ treatment did not induce a significant change in Bax expression, whereas it caused a significant decrease in Bcl-2 expression and increased expression of cleaved caspase-3 and cleaved PARP. These findings are consistent with our previously reported in vitro results [17,18] and suggest that apoptosis induced by H_2_O_2_ under in vitro conditions is closely related to the decrease in Bcl-2 expression. This pattern is consistent with the intrinsic apoptosis pathway. According to previous studies, Bax is a pro-apoptotic factor that promotes caspase-3 activation and PARP cleavage by inducing cytochrome c release through the facilitation of mitochondrial outer membrane permeabilization (MOMP), whereas Bcl-2 is a well-known anti-apoptotic protein that inhibits Bax activity [57,58]. In line with the intrinsic apoptotic pathway, numerous previous studies have used the Bax/Bcl-2 ratio as a key indicator in Western blot analyses to evaluate apoptosis, rather than relying solely on changes in the expression of Bax or Bcl-2 individually [59,60].

In this context, the present study interpreted the results by comprehensively considering key apoptosis-related factors, including the balance between Bax and Bcl-2, caspase-3 and PARP, rather than relying on changes in Bax as a single indicator. In this study, neither statistically significant changes nor dose-dependent patterns were observed in Bax expression in the RMB, Tax-G, and Tax-A treatment groups. In contrast, expression of the Bcl-2 was significantly restored, while the expression of cleaved caspase-3 and cleaved PARP was suppressed. These results suggest that RMB, Tax-G, and Tax-A may inhibit mitochondria-mediated apoptosis under oxidative stress conditions. In particular, the Tax-A treatment group showed the strongest recovery of Bcl-2 expression and suppression of apoptosis markers among the three substances, indicating that Tax-A may exhibit relatively strong anti-apoptotic activity under oxidative stress conditions. However, the present study has limitations in that apoptosis-related markers were primarily evaluated at the protein level. Therefore, further research incorporating mRNA-level analyses will be required to more clearly elucidate the regulatory mechanisms underlying oxidative stress-induced muscle cell damage.

In the muscle atrophy model, the DEX-treated group showed a reduced myotube diameter, along with increased expression of the muscle protein degradation factors Atrogin-1 and MuRF1, and suppressed expression of the muscle differentiation factors MyoD and Myogenin. Additionally, it significantly reduced the phosphorylation ratios of Akt, mTOR, and FoxO3α. These results are consistent with the well-established mechanism, where DEX inhibits the Akt/mTOR pathway, a muscle synthesis signal, while activating the FoxO pathway, a muscle atrophy signal, leading to activation of the ubiquitin–proteasome system and suppression of muscle synthesis regulators [6,14,15]. The RMB, Tax-G, and Tax-A treatment groups inhibited the expression of Atrogin-1 and MuRF1, restoring the expression of MyoD and Myogenin, and reinstated the phosphorylation ratios of Akt, mTOR, and FoxO3α. These results were consistent with the significant recovery of myotube diameter observed following treatment with RMB, Tax-G, and Tax-A, suggesting that these substances may exert muscle-protective effects by inhibiting protein degradation and promoting protein synthesis. When comparing the biomarker analysis results among treatments, the recovery of myotube diameter was most prominent in the Tax-A group, whereas for muscle synthesis and degradation factors, the Tax-G group showed effects comparable to or higher than those of the aglycone at the same concentration (50 μM). However, the present study has limitations in that the regulation of the Akt/mTOR and FoxO signaling pathways was interpreted based solely on changes in phosphorylation, without functional or pharmacological validation. Therefore, further studies are required to elucidate the mechanistic causal relationship between these signaling pathways and the observed muscle-protective effects.

Tax-A has attracted attention in the pharmaceutical, health care product, and food industries due to its strong antioxidant and anti-inflammatory activities, but its use has been limited by low bioavailability [61]. To overcome this, various developments in drug delivery systems, including nanoparticles and water-soluble derivatives, have been pursued [46,47,62]. Meanwhile, flavonoid glycosides are known to exhibit higher bioavailability, greater in vivo persistence, and more stable forms in nature compared with aglycones [45]. Therefore, identifying taxifolin glycosides offers major advantages in terms of efficiency and economic feasibility for the development of useful materials. In this regard, taxifolin-3-O-arabinopyranoside, used in this study, is abundantly present in *R. mucronulatum* extract and exhibited bioactivity comparable to that of Tax-A. Accordingly, it represents a promising compound of substantial value.

In conclusion, this study is significant in that it comprehensively evaluated the effects of RMB, Tax-G, and Tax-A on apoptosis and muscle atrophy, the key pathological mechanisms of sarcopenia. The findings provide important insights into the potential of natural product-based candidates for mitigating cellular mechanisms associated with sarcopenia. In particular, the finding that Tax-G, which is advantageous in terms of stability and bioavailability, exhibited anti-apoptotic and muscle-protective effects comparable to Tax-A supports its potential as a bioactive compound. However, this study is limited to an in vitro-based preliminary investigation and lacks a positive control group. To address this limitation, the effects of the test substances were evaluated through statistical comparisons based on normal and negative control groups to ensure objectivity and scientific validity. Furthermore, based on these in vitro findings, further studies using in vivo animal models relevant to sarcopenia will be required. Such follow-up studies will provide important evidence for assessing the in vivo relevance of the present findings.

## 4. Materials and Methods

### 4.1. Plant Materials

This study was conducted using *R. mucronulatum* branch extract (RMB), extracted on a pilot scale. The sample is stored at the Department of Forest Biomaterials Engineering, Kangwon National University. *R. mucronulatum* branches were purchased from the Seoul Yakryeong Medicine Market and used after being certified by Professor Choi (Department of Forest Biomaterials Engineering, Kangwon National University).

### 4.2. Pilot-Scale Extraction of Branch of Rhododendron mucronulatum

Based on the extraction conditions established in the lab scale and semi-pilot-scale stages, 300 kg of *R. mucronulatum* branches were extracted with 50% edible ethanol at 80 °C for 8 h in the pilot-scale stage and then concentrated. 23 kg of *R. mucronulatum* branch concentrate (yield 7.7%) was obtained. 17 kg of *R. mucronulatum* branch concentrate was freeze-dried to recover 9 kg of powder (yield 4%). 10% dextrin was added to this powder to obtain 10 kg of *R. mucronulatum* branch extract powder (RMB, Lot. No. DJTH-05572). The extraction process was performed at DanjoungBio Co., Ltd. (Wonju, Republic of Korea).

### 4.3. Phytochemical Analysis

#### 4.3.1. Standard Compound

**Figure 16:**
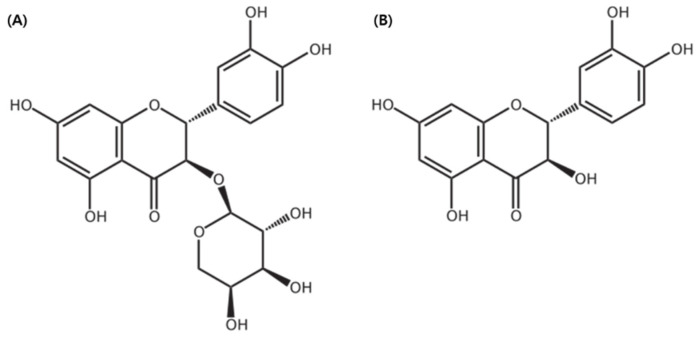
The structure of (**A**) taxifolin-3-O-arabinopyranoside and (**B**) taxifolin aglycone. The chemical structure was illustrated using ChemDraw Ultra 7.0 (CambridgeSoft, Cambridge, MA, USA).

The standard compounds—Taxifolin-3-O-arabinopyranoside and taxifolin aglycon—were stored at the Department of Forest Biomaterials Engineering, Kangwon National University. These compounds had previously been isolated, purified, and structurally identified from *R. mucronulatum* root extracts, as described in the works of Kim et al. (2024) [31] and Park et al. (2022) [40], respectively (Figure 16).

#### 4.3.2. Qualitative Analysis of RMB (TLC)

Thin-layer chromatography (TLC) was performed for qualitative analysis of the standard compounds. The standards and each sample were each dissolved in methanol to prepare analytical samples at a concentration of 1 mg/mL. Each analytical sample was spotted onto a silica gel plate (TLC aluminum sheet Silica gel 60 F_254_, Sigma-Aldrich, St. Louis, MO, USA) and developed using a mobile phase consisting of chloroform:methanol:water at a 70:30:4 (*v*/*v*/*v*) ratio and chloroform:methanol at a 5:1 (*v*/*v*). After the silica gel plate was completely dried, it was detected using three spray reagents (10% H_2_SO_4_, *ρ*-anisaldehyde–H_2_SO_4_, FeCl_3_) and ultraviolet light at 254 nm.

#### 4.3.3. HPLC Quantitative Analysis and LC-MS/MS Analysis of RMB

HPLC analysis was performed using a Waters 2695 Separation Module and a Waters 2487 Dual-λ Absorbance Detector (Waters, Milford, MA, USA). Chromatographic separation was carried out on an OptimaPak C18 analytical column (250 × 4.6 mm, 5 μm) (Chiral Technology Korea Co., Ltd., Daejeon, Republic of Korea), which was fitted with a Phenomenex KJ0-4282 guard column. The column temperature was maintained at 30 °C. The mobile phase consisted of 1% formic acid in water (A) and acetonitrile (B). The flow rate was set at 1 mL/min, the injection volume was 20 μL, and a detection wavelength set at 280 nm. An elution gradient was employed as follows: 0–0 min, 10% B; 0–20 min, 35% B; 20–25 min, 100% B; 25–35 min, 10% B; 35–40 min, 10% B.

For quantitative analysis, each standard compound—Tax-G, Tax-A—was dissolved in methanol and then diluted to a range of 1–600 μg/mL and 1–250 μg/mL, respectively. Calibration plots for Tax-G, Tax-A were constructed using the peak areas (y) obtained from 7 different concentration solutions (x) to establish each standard curve. The formulas used to calculate the LOD and LOQ are as follows:

LOD (Limit of Detection, μg/mL) = 3.3 × SE × √N/S(1)

LOQ (Limit of Quantitation, μg/mL) = 10 × SE × √N/S(2)

where ‘SE’ = Standard Error of the y-intercept, ‘N’ = the number of tests, and ‘S’ = slope of the calibration curve.

All analytical samples were dissolved in methanol at a concentration of 1000 μg/mL and filtered through a 0.2 μm syringe filter to prepare the test solutions. To ensure repeatability, each sample was analyzed with triplicate injections, and the retention times were verified by comparison with authentic standards. Quantitative analysis was conducted using the regression equation derived from the standard calibration curve. This analysis was subsequently validated using LOD, which indicates the lowest concentration at which the presence of the target compound can be clearly identified, and LOQ, which indicates the lowest concentration that ensures the reliability of the results.

The molecular weight of each peak was confirmed through LC-MS/MS analysis. The analytical equipment used was AB SCIEX (QTRAP 4500, Framingham, MA, USA), and the analytical conditions were identical to those used for HPLC.

#### 4.3.4. NMR Analysis

For NMR analysis, Bruker’s 700 MHz Avance III 700 (Bruker Biospin GmbH, Rheinstetten, Germany) model owned by the Gyeonggi Bio Center was used. MeOH-*d*_4_ was used as a solvent, and the structures of compound **1** and compound **2** obtained through separation and purification using ^1^H-NMR (700 MHz) and ^13^C-NMR (176 MHz) analysis were confirmed.

### 4.4. Separation and Purification of Taxifolin Glycoside and Taxifolin Aglycon

Separation and purification of taxifolin-3-O-arabinopyranoside (Tax-G) and taxifolin aglycone (Tax-A) were performed through MPLC (Yamazen Smart Flash AI-580S system Medium Pressure Liquid Chromatography; Yamazen Corp., Osaka, Japan). Silica gel L size (Yamazen Corp., Osaka, Japan) was used as the injection column. Silica gel column 2 L size (40 µm, 60 Å, 3.0 × 20.0 cm, 55 g) (Yamazen Corp., Osaka, Japan) was used for the primary purification, and ODS-SM column 2 L size (50 µm, 120 Å, 3.0 × 20.0 cm, 51 g) (Yamazen Corp., Osaka, Japan) was used from the secondary purification onward. The flow rate was 20 mL/min, the wavelength was confirmed at 280 nm, and the injection volume was 1 mL.

#### 4.4.1. Separation and Purification of Tax-G

Tax-G was isolated by modifying the method of Kim et al. (2024) [31]. 300 g of the RMB was dissolved in distilled water and combined with ethyl acetate at an 8:10 ratio (*v*/*v*). The mixture fractionated with ethyl acetate using a separatory funnel. After fractionation, the ethyl acetate layer was filtered with filter paper and then concentrated under reduced pressure to obtain 31.29 g (yield 10.43%) in powder form (RMB RF1).

20 g of RMB RF1 was dissolved in methanol at a concentration of 0.2 g/mL, and separation and purification were performed through MPLC. The mobile phase of primary separation and purification was mixed with chloroform:methanol:water in a ratio of 70:30:4 (*v*/*v*/*v*) and proceeded with an isocratic system. The analysis time was set to 17 min. The primary purified product was concentrated under reduced pressure to obtain 5.20 g of powder. Afterwards, to secure a single compound of high purity, secondary separation and purification were performed using an ODS column. The mobile phase was water and methanol in a gradient system; 0 min, 0% MeOH; 0–10 min, 25% MeOH; 10–40 min, 40% MeOH; 40–43 min, 100% MeOH; 43–49 min, 100% MeOH; 49–54 min, 0% MeOH; 54–62 min, 0% MeOH.

#### 4.4.2. Separation and Purification of Tax-A

RMB was hydrolyzed in an eco-friendly manner using food-grade enzymes according to the method described by Park et al. (2022) [40]. Specifically, 10 g of RMB was dissolved in distilled water 900 mL, and Pectinex XXL and Ultra SP-L (Novozymes Co., Ltd., Bagsvaerd, Denmark) (50 mL each, total 100 mL, 10%, *w*/*w*) were added. The mixture was stirred at 50 °C for 24 h. To inactivate and remove the enzymes, the reaction was heated for 10 min at about 85 °C, followed by centrifugation and filtration. The filtrate was then fractionated with ethyl acetate, and the ethyl acetate layer was concentrated under reduced pressure (RMB RF2).

1.1 g of RMB RF2 was dissolved in methanol at a concentration of 0.2 g/mL, and separation and purification were performed by Silica gel column with CM solution (chloroform:methanol = 5:1) in an isocratic system. The analysis time was set to 20 min. After that, the isolating fraction was concentrated into a powder. Then, the powder (0.54 g) was dissolved in MeOH at a concentration of 0.1 g/mL, and repeated purification was performed using an ODS column with water and methanol in a gradient system; 0 min, 0% MeOH; 0–15 min, 32% MeOH; 15–21 min, 35% MeOH; 21–41 min, 40% MeOH; 41–44 min, 100% MeOH; 44–52 min, 100% MeOH; 52–55 min, 0% MeOH; 55–63 min, 0% MeOH.

### 4.5. Cell Culture and Treatments

C2C12 cells used in this study were obtained from the American Type Culture Collection (ATCC, Manassas, VA, USA). Cells were maintained in a humidified incubator with 5% CO_2_ and 95% air at 37 °C in Dulbecco’s Modified Eagle Medium (DMEM) supplemented with 10% fetal bovine serum (FBS), 100 U/mL penicillin, and 100 μg/mL streptomycin. When cells reached approximately 80% confluence, the monolayer was washed with phosphate-buffered saline (PBS, pH 7.4) and subcultured using trypsin–EDTA (2.65 mM). The culture medium was replaced every two days.

The test substances used in this study were RMB, Tax-G, and Tax-A. All substances were applied at concentrations that did not exhibit cytotoxicity under normal conditions.

All samples were dissolved in dimethyl sulfoxide (DMSO) and co-treated with the model-inducing agents. The final DMSO concentration was maintained below 0.1%, a level that has been reported to exert no significant effects on cell viability or major muscle-related parameters [63,64,65,66].

#### 4.5.1. Apoptosis Induction and Treatment

C2C12 cells were seeded in 24-well plates at 5 × 10^4^ cells/well and cultured for 24 h. Subsequently, oxidative stress-induced apoptosis was induced by treatment with 100 μM H_2_O_2_, as described in previous studies [17,18,19,20,59,67,68].

#### 4.5.2. Muscle Atrophy Induction and Treatment

C2C12 cells were seeded at a density of 5 × 10^4^ cells per well in 24-well plates and cultured for 24 h. To induce differentiation into myotubes, C2C12 cells were incubated for 4 days in differentiation medium consisting of DMEM supplemented with 2% horse serum (HS), which was refreshed every two days. To induce myotube atrophy, cells were treated with 5 μM dexamethasone (DEX), as described in previous studies [17,18,19,20,69,70,71].

### 4.6. Cell Viability Assay

#### 4.6.1. Measurement of C2C12 Myoblast Viability Using MTT Assay

The cell viability of C2C12 cells was measured using the MTT assay method [72]. C2C12 cells were seeded at 5 × 10^4^ cells/well in 24-well plates and cultured for 24 h. The culture medium was then replaced with medium containing various concentrations of the test substances, and the cells were further incubated for 48 h. Subsequently, the medium was replaced with 1 mg/mL MTT solution, and the cells were incubated for an additional 2 h. The formazan crystals formed in viable cells were dissolved in isopropanol, and absorbance was measured at 570 nm to determine cell viability.

#### 4.6.2. Measurement of Protective Effects Against H_2_O_2_-Induced Myoblast Damage

C2C12 cells were seeded at 5 × 10^4^ cells/well in 24-well plates and cultured for 24 h. To induce apoptosis, the cells were treated with 100 μM H_2_O_2_ together with the three test substances at various concentrations and incubated for 48 h. After 48 h of incubation, cell viability was measured using the MTT assay as described above.

#### 4.6.3. Measurement of Protective Effects Against DEX-Induced Myotube Damage

C2C12 cells were seeded at 5 × 10^4^ cells/well in 24-well plates and cultured for 24 h. As described above, to induce muscle atrophy, C2C12 cells were differentiated into myotubes and then treated with 5 μM dexamethasone together with various concentrations of the test substances for 24 h. After 24 h of incubation, cell viability was measured using the MTT assay as described above.

### 4.7. Effects of Rhododendron mucronulatum Branch Extract (RMB), Taxifolin-3-O-Arabinopyranoside (Tax-G) and Taxifolin (Tax-A) on Muscle Apoptosis Biomarkers

#### 4.7.1. Evaluation of Apoptosis in H_2_O_2_ Induced Myoblasts

To evaluate the effects of the three test substances on H_2_O_2_-induced apoptosis, C2C12 cells were seeded at 5 × 10^4^ cells/well in 24-well plates and cultured for 24 h. The cells were then treated with 100 μM H_2_O_2_ together with the three test substances at various concentrations and incubated for 48 h. The degree of apoptosis in myocytes was measured using a Cellular DNA Fragmentation ELISA kit (Sigma-Aldrich), which detects 5′-bromo-2′-deoxy-uridine (BrdU)-labeled DNA, according to the manufacturer’s instructions.

#### 4.7.2. Western Blot Analysis of Apoptosis Biomarkers in C2C12 Myoblasts

C2C12 cells were seeded at a density of 1 × 10^6^ cells per 100 mm dish and cultured for 24 h. The cells were then treated with 100 μM H_2_O_2_ together with various concentrations of the three test substances for an additional 24 h. For protein extraction, cells were lysed in buffer (20 mmol/L Hepes, pH 7.5; 150 mmol/L NaCl; 1% Triton X-100; 1 mmol/L EDTA; 1 mmol/L EGTA; 100 mmol/L NaF; 10 mmol/L sodium pyrophosphate; 1 mmol/L Na_3_VO_4_; 20 μg/mL aprotinin; 10 μg/mL antipain; 10 μg/mL leupeptin; 80 μg/mL benzamidine HCl; and 0.2 mmol/L PMSF), and total cell lysates were collected by centrifugation. Protein concentrations were determined using a BCA Protein Assay Kit (Thermo Scientific). Equal amounts of protein (50 μg) were separated by 10% SDS-PAGE and transferred onto PVDF membranes (Millipore, Burlington, MA, USA). Membranes were blocked with 5% skim milk in TBST (20 mmol/L Tris-HCl, pH 7.5; 150 mmol/L NaCl; 0.1% Tween 20) for 1 h, and then incubated with primary antibodies against Bax, Bcl-2, cleaved caspase-3, cleaved PARP, and β-actin (Cell Signaling Technology, Beverly, MA, USA) overnight at 4 °C or for 1 h at room temperature. After washing, membranes were incubated with HRP-conjugated secondary antibodies (anti-rabbit or anti-mouse IgG) for 1 h. Protein bands were visualized by enhanced chemiluminescence using Immobilon™ Western Chemiluminescent HRP Substrate (Millipore) and quantified with an ImageQuant™ LAS 500 imaging system (GE Healthcare Bio-Sciences AB, Uppsala, Sweden). β-actin was used as an internal control. The band intensities of the target proteins were normalized to the β-actin and expressed relative to the control group.

### 4.8. Effects of Rhododendron mucronulatum Branch Extract (RMB), Taxifolin-3-O-Arabinopyranoside (Tax-G) and Taxifolin (Tax-A) on Muscle-Synthesis- and Muscle-Degradation Biomarkers

#### 4.8.1. Measurement of Myotube Diameter

C2C12 cells were seeded at 5 × 10^4^ cells/well in 24-well plates containing cover glasses and cultured for 24 h. As described above, after differentiation into myotubes, 5 μM DEX was added to induce muscle atrophy, and the three test substances were treated for 24 h. After incubation, the medium was removed and the cells were washed with PBS, followed by fixation with 4% paraformaldehyde and permeabilization with 0.1% Triton X-100. After blocking with 5% BSA/TBST, the cells were incubated with a primary antibody (MYH7; Santa Cruz, Dallas, TX, USA). Subsequently, staining was performed with a secondary antibody (anti-mouse IgG–Alexa-594; Thermo Fisher Scientific, Waltham, MA, USA), and counterstaining was carried out with 4′,6-diamidino-2-phenylindole (DAPI; Sigma-Aldrich, St. Louis, MO, USA). Fluorescent cell images (five images per group) were captured using a fluorescence microscope (AxioImager, Carl Zeiss, Jena, Germany) at 20× magnification. Ten myotubes per image were randomly selected in each microscopic field, and the diameter at the thickest part of each myotube was measured using ImageJ software (National Institutes of Health, Bethesda, MD, USA, version 1.54).

#### 4.8.2. Real-Time Reverse Transcription Polymerase Chain Reaction (RT-PCR) Analysis

C2C12 cells were seeded at 5 × 10^4^ cells/well in 24-well plates and cultured for 24 h. As described above, after differentiation into myotubes, muscle atrophy was induced by adding 5 μM DEX, and the three test substances were treated for 24 h. The cells were then collected, and total RNA was isolated using the RNeasy^®^ Plus Mini Kit (QIAGEN, Valencia, CA, USA). The quantity and purity of total RNA were measured using a micro-volume spectrophotometer (BioSpec-nano, SHIMADZU, Kyoto, Japan), and only RNA with an OD_260_/_280_ ratio greater than 1.8 was used for experiments.

cDNA was synthesized from 2 μg of total RNA using the HyperScript™ RT Master Mix Kit (GeneAll Biotechnology, Seoul, Republic of Korea). Real-time PCR was performed with the Rotor-Gene 300 PCR system (Corbett Research, Mortlake, Australia) using the QuantiNova SYBR Green PCR Kit (QIAGEN, Valencia, CA, USA). Information on the primer used in the experiments is shown in Table 4. Quantitative analysis of gene expression was carried out using the Rotor-Gene 6000 Series System Software (Corbett Research, Mortlake, NSW, Australia, version 6). Ct values were obtained using a real-time PCR system, and relative expression levels were calculated using the 2^−ΔΔCt^ method. The Ct values of target genes were normalized to those of a reference gene (GAPDH). All results were expressed as percentages relative to the control group.

#### 4.8.3. Western Blot Analysis of Muscle Synthesis- and Muscle Degradation-Related Proteins in C2C12 Myotubes

C2C12 cells were seeded at 2 × 10^6^ cells in 100 mm dishes and cultured for 24 h. As described above, after differentiation into myotubes, muscle atrophy was induced by adding 5 μM DEX, and the three test substances were treated for 24 h. After incubation, Western blot analysis was performed as previously described. In this analysis, antibodies against Atrogin-1, MuRF1 (Santa Cruz, Santa Cruz, CA, USA), MyoD1, Myogenin (Abcam, Cambridge, MA, USA), phospho-Akt (Ser473), Akt, phospho-mTOR (Ser253), mTOR, phospho-FoxO3 (Ser256), FoxO3, phospho-FoxO3 (Ser253), FoxO3, and β-actin (Cell Signaling Technology) were used. The detected protein bands were visualized by enhanced chemiluminescence using Luminata™ Forte Western HRP Substrate (Millipore). Protein expression was quantified using an ImageQuant™ LAS 500 imaging system (GE Healthcare Bio-Sciences AB, Billerica, MA, USA). β-actin was used as an internal control. The band intensities of the target proteins were normalized to the β-actin and expressed relative to the control group.

### 4.9. Statistical Analysis

All analysis values are expressed as the mean ± S.E.M. The collected results were analyzed using the GraphPad Prism 5.0 software (GraphPad Software, San Diego, CA, USA). Student’s t-test and a one-way analysis of variance (ANOVA) were used to compare differences between the control group and the test substance treatment group. It was considered statistically significant only when *p* < 0.05.

## 5. Conclusions

In this study, the anti-apoptotic and muscle atrophy-protective effects of *Rhododendron mucronulatum* branch extract (RMB) and its active compounds, taxifolin-3-O-arabinopyranoside (Tax-G) and taxifolin (Tax-A), were evaluated using C2C12 cell models. Phytochemical analysis confirmed the efficiency and industrial feasibility of the pilot-scale extraction process for *R. mucronulatum* branches, which, compared to the previously used root part, provides sustainable raw material acquisition and economic advantages for industrial application. Additionally, the enzymatic hydrolysis process indicated the potential for producing the high value-added compound Tax-A in an eco-friendly manner.

In the in vitro experiments, RMB, Tax-G, and Tax-A all inhibited apoptosis induced by H_2_O_2_ through increased Bcl-2 expression and decreased cleaved caspase-3 and cleaved PARP. Furthermore, in the dexamethasone-induced muscle atrophy model, they were associated with the regulation of the muscle protein synthesis- and degradation-related markers, as well as the recovery of myotube diameter, suggesting muscle-protective effects at the cellular level.

These findings provide insights into the potential of RMB, Tax-G, and Tax-A as natural product-based candidates for modulating cellular mechanisms associated with sarcopenia. In particular, Tax-G showed anti-apoptotic and muscle-protective effects comparable to Tax-A while exhibiting no distinct cytotoxicity. Moreover, Tax-G can be abundantly obtained from *R. mucronulatum* extract without additional processing, providing advantages not only in safety but also in material efficiency.

However, this study is limited to in vitro cell models. Therefore, further studies using in vivo animal models and pharmacokinetic analyses are required to elucidate their actual efficacy and mechanisms in living systems.

## Figures and Tables

**Table 1:** Effect of *Rhododendron mucronulatum* branch extract (RMB) on muscle degradation-and muscle synthesis-related gene expression (% of control group).

DEX (5 μM)	RMB (μg/mL)	mRNA			
		Atrogin-1	MuRF1	MyoD1	Myogenin
−	−	6.36 ± 1.02	8.49 ± 0.85	344.21 ± 47.31	288.19 ± 28.07
+	−	100 ± 11.56 **	100 ± 10.46 ***	100 ± 7.05 ***	100 ± 10.23 ***
+	50	88.46 ± 11.37	80.28 ± 18.88	125.65 ± 9.29	135.89 ± 24.66
+	100	50.59 ± 6.68 ^##^	64.95 ± 5.06 ^#^	194.11 ± 21.03 ^##^	181.69 ± 46.27
+	200	52.27 ± 9.07 ^##^	35.62 ± 6.73 ^###^	188.84 ± 18.80 ^##^	170.43 ± 18.96 ^##^

DEX: dexamethasone; RMB: *Rhododendron mucronulatum* branch extract. Values are expressed as mean ± S.E.M. (n = 6). The target mRNA’s expression was normalized to that of GAPDH, and values represent relative quantification. ** *p* < 0.01 and *** *p* < 0.001 indicate significantly different from the [DEX (−)/RMB (−)] group. ^#^ *p* < 0.05, ^##^ *p* < 0.01, and ^###^ *p* < 0.001 indicate significantly different from the [DEX (+)/RMB (−)] group.

**Table 2:** Effect of Taxifolin-3-O-arabinopyranoside (Tax-G) on muscle-degradation- and muscle-synthesis-related gene expression (% of control group).

DEX (5 μM)	Tax-G (μM)	mRNA			
		Atrogin-1	MuRF1	MyoD1	Myogenin
−	−	5.38 ± 0.42	4.86 ± 0.36	370.58 ± 55.35	270.98 ± 23.47
+	−	100 ± 17.32 ***	100 ± 8.15 ***	100 ± 19.46 ***	100 ± 10.16 ***
+	10	71.52 ± 11.93	82.29 ± 5.83	136.45 ± 13.77	144.47 ± 12.39 ^#^
+	50	66.12 ± 5.47	65.72 ± 8.07 ^#^	214.08 ± 33.48 ^#^	198.08 ± 23.77 ^##^
+	100	50.87 ± 4.15 ^#^	55.61 ± 3.49 ^###^	305.36 ± 54.58 ^##^	248.93 ± 25.62 ^###^

DEX: dexamethasone; Tax-G: Taxifolin-3-O-arabinopyranoside. Values are expressed as mean ± S.E.M. (n = 6). The target mRNA expression was normalized to that of GAPDH, and values represent relative quantification. *** *p* < 0.001 indicates significantly different from the [DEX (−)/Tax-G (−)] group. ^#^ *p* < 0.05, ^##^ *p* < 0.01, and ^###^ *p* < 0.001 indicate significantly different from the [DEX (+)/Tax-G (−)] group.

**Table 3:** Effect of Taxifolin (Tax-A) on muscle-degradation- and muscle-synthesis-related gene expression (% of control group).

DEX (5 μM)	Tax-A (μg/mL)	mRNA			
		Atrogin-1	MuRF1	MyoD1	Myogenin
−	−	5.96 ± 0.23	5.78 ± 0.76	315.42 ± 46.51	309.10 ± 25.80
+	−	100 ± 5.96 **	100 ± 11.46 ***	100 ± 22.68 **	100 ± 17.27 **
+	5	76.12 ± 6.11 ^#^	100.94 ± 11.54	140.07 ± 11.58	150.26 ± 7.34 ^#^
+	10	70.86 ± 5.31 ^##^	72.64 ± 3.96 ^#^	201.17 ± 20.49 ^##^	181.10 ± 17.91 ^##^
+	50	59.38 ± 6.08 ^###^	62.65 ± 5.40 ^#^	217.63 ± 22.23 ^##^	197.65 ± 21.57 ^##^

DEX: dexamethasone; Tax-A: Taxifolin. Values are expressed as mean ± S.E.M. (n = 6). The target mRNA expression was normalized to that of GAPDH and values represent relative quantification. ** *p* < 0.01 and *** *p* < 0.001 indicate significantly different from the [DEX (−)/Tax-A (−)] group. ^#^ *p* < 0.05, ^##^ *p* < 0.01, and ^###^ *p* < 0.001 indicate significantly different from the [DEX (+)/Tax-A (−)] group.

**Table 4:** Specific primer sequences for RT-PCR.

mRNA	Primer Sequences		Genebank No.
Atrogin-1	Forward	5′-GCCCTCCACACTAGTTGACC-3′	NM_026346.3
	Reverse	5′-GACGGATTGACAGCCAGGAA-3′	
MuRF1	Forward	5′-GAGGGCCATTGACTTTGGGA-3′	NM_001039048.2
	Reverse	5′-TTTACCCTCTGTGGTCACGC-3′	
MyoD1	Forward	5′-GCACTACAGTGGCGACTCAGAT-3′	NM_010866.2
	Reverse	5′-TAGTAGGCGGTGTCGTAGCCAT-3′	
Myogenin	Forward	5′-CCATCCAGTACATTGAGCGCCT-3′	NM_031189.2
	Reverse	5′-CTGTGGGAGTTGCATTCACTGG-3′	
GAPDH	Forward	5′-TGGGTGTGAACCATGAGAAG-3′	NM_008084.3
	Reverse	5′-GCTAAGCAGTTGGTGGTGC-3′	

## Data Availability

The original contributions presented in this study are included in the article/Supplementary Material. Further inquiries can be directed to the corresponding author.

## Supplementary Materials

The following supporting information can be downloaded at: https://www.mdpi.com/article/10.3390/ijms27020570/s1.

## Abbreviations

The following abbreviations are used in this manuscript:

RMB	*Rhododendron mucronulatum* branch extract
Tax-G	Taxifolin-3-O-arabinopyranoside
Tax-A	Taxifolin
RF	Rich Fraction
ICD	International classification of diseases
NAFLD	Non-alcoholic fatty liver disease
TLC	Thin-layer chromatography
HPLC	High-performance liquid chromatography
MPLC	Medium pressure liquid chromatography
LC-MS/MS	Liquid chromatography–tandem mass spectrometry
NMR	Nuclear magnetic resonance
MTT	3-(4,5-dimethylthiazol-2-yl)-2,5-diphenyltetrazolium bromide
Bax	Bcl-2-Associated X Protein
Bcl-2	B-cell Lymphoma-2
PARP	Poly(ADP-Ribose) Polymerase
RT-PCR	Real-time polymerase chain reaction
GAPDH	Glyceraldehyde-3-phosphate Dehydrogenase
MuRF1	Muscle RING-finger Protein 1
MyoD	Myoblast Determination Protein
Akt	Protein Kinase B
mTOR	Mammalian Target of Rapamycin
FoxO3α	Forkhead Box O3α
IGF-1	Insulin-like Growth Factor-1
ROS	Reactive oxygen species
DEX	Dexamethasone
MOMP	Mitochondria outer membrane pore
S.E.M.	Standard Error of the Mean
