# Supplementary material for: In Vitro Study on the Effects of Rhododendron mucronulatum Branch Extract, Taxifolin-3-O-Arabinopyranoside and Taxifolin on Muscle Loss and Muscle Atrophy in C2C12 Murine Skeletal Muscle Cells
Source: Int J Mol Sci. 2026 Jan 6;27(2):570. doi: 10.3390/ijms27020570 (PMC12841282; doi:10.3390/ijms27020570)
Supplement: Supplementary file 1 [file ijms-27-00570-s001.zip › ijms-4050765-supplementary.pdf]

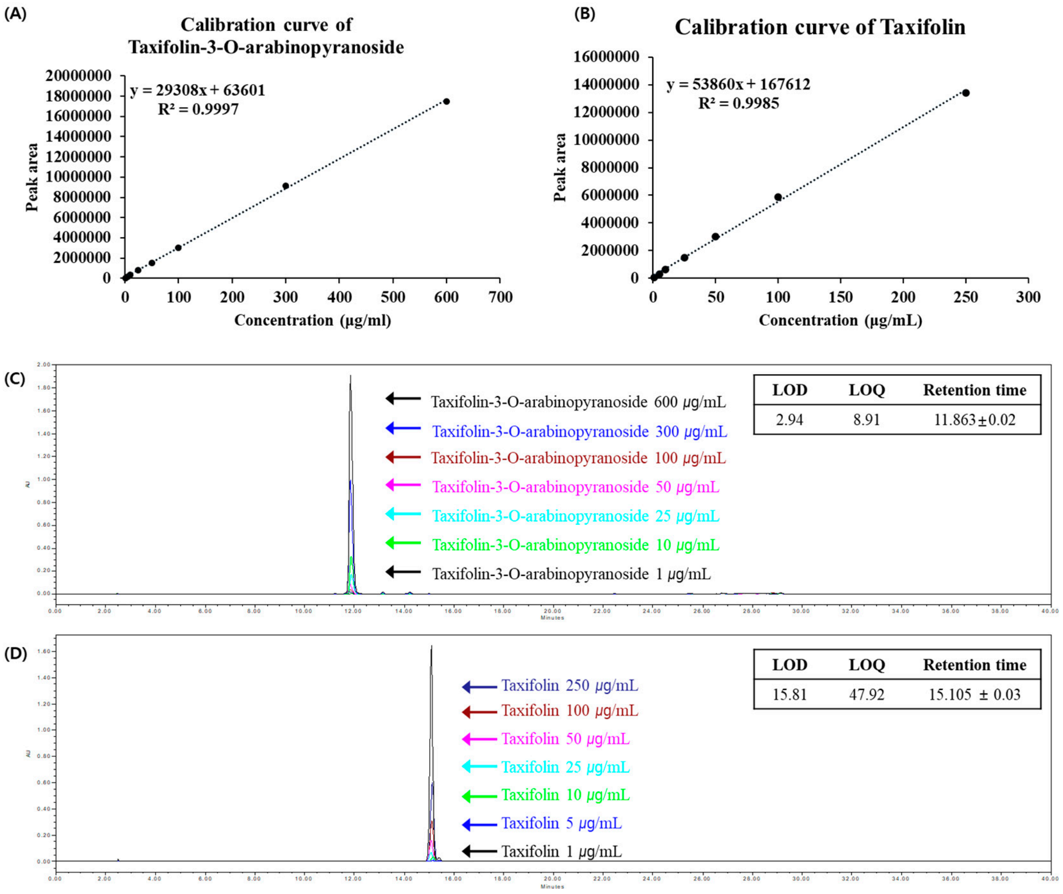

Figure S1. Calibration curves and regression equations for **(A)** Taxifolin-3-O-arabinopyranoside (Tax-G), **(B)** Taxifolin aglycone (Tax-A). Representative HPLC chromatograms of **(C)** Tax-G at concentrations of 1-600 μg/mL and **(D)** Tax-A at concentrations of 1-250 μg/mL. The table on the right presents the limit of detection (LOD), limit of quantification (LOQ) and retention time for each compound.

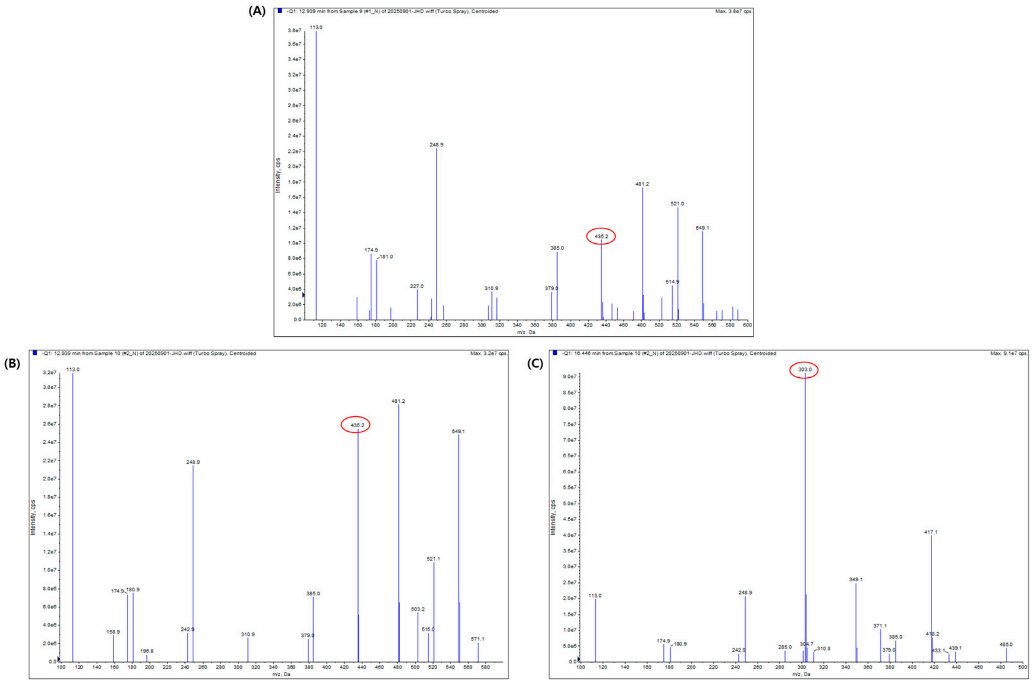

Figure S2. Negative mode LC-MS/MS spectra of RMB and enzyme hydrolyzed RMB. **(A)** MS/MS spectrum of the peak at 12.939 min in RMB (435.2 m/z)**, (B)** MS/MS spectrum of the peak at 12.939 min in RF2 (435.2 m/z)**, (C)** MS/MS spectrum of the peak at 15.446 min in RF2 (303.0 m/z).

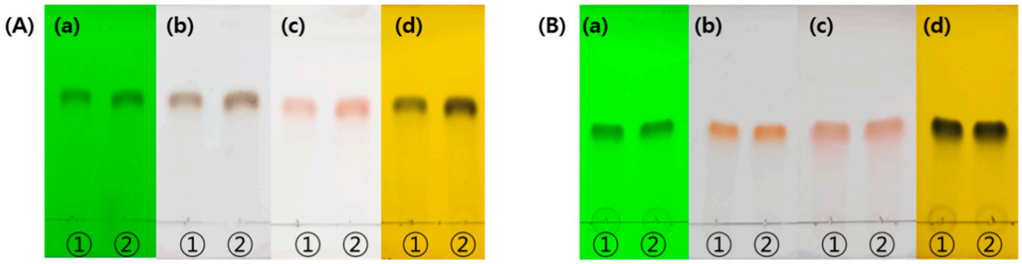

Figure S3. TLC monitoring of (**A**) ① taxifolin-3-O-arabinopyranoside and ② compound 1, (**B**) ①taxifolin aglycone and ② compound 2. (a) UV 254 nm, (b) 10% H_2SO_4, (c) *ρ*-anisaldehyde H_2SO_4, and (d) $FeCl_3$. The eluent system employed was (A) chloroform: methanol = 5: 1 and (B) chloroform: methanol: water = 70: 30: 4 (v/v/v).

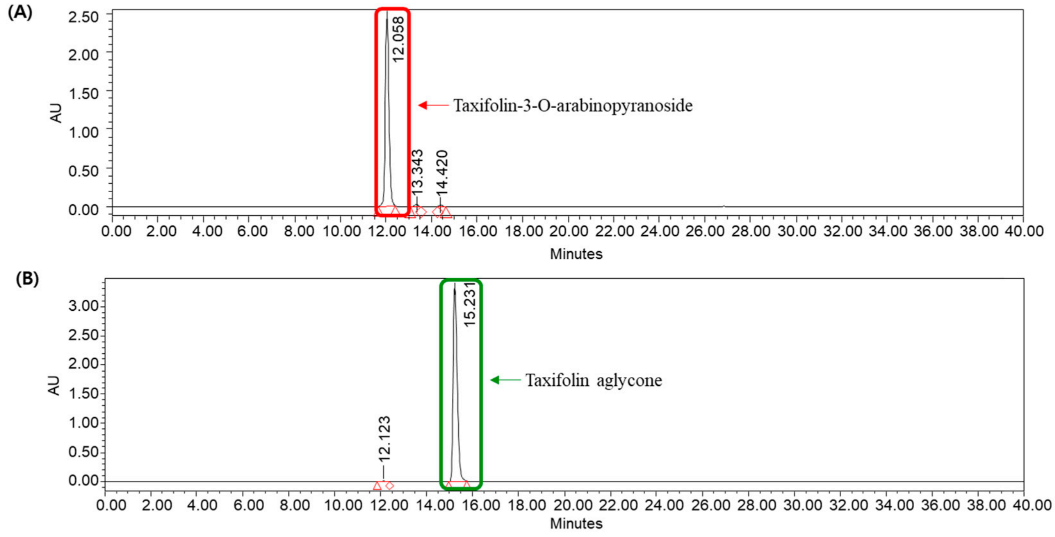

Figure S4. HPLC chromatogram of (**A**) compound 1 (Tax-G; 1,000 μg/mL, purity: 98.21%) and (**B**) compound 2 (Tax-A; 1,000 μg/mL, purity: 99.68%).

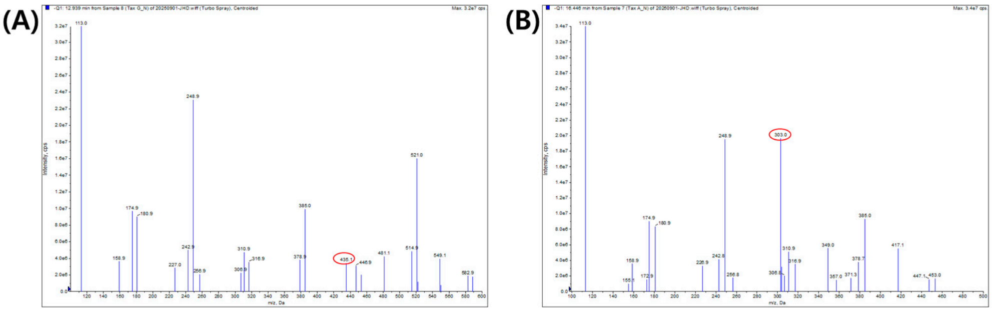

Figure S5. Negative mode LC-MS/MS spectra of (**A**) compound 1 (Tax-G; 435.1 m/z) and (**B**) compound 2 (Tax-A; 303.0 m/z).
